# Supplementary material for: Reference-based comparison of adaptive immune receptor repertoires
Source: Cell Rep Methods. 2022 Aug 22;2(8):100269. doi: 10.1016/j.crmeth.2022.100269 (PMC9421535; doi:10.1016/j.crmeth.2022.100269)
Supplement: Document S1. Figures S1–S6 and Tables S1 and S2 [file mmc1.pdf]

Cell Reports Methods, Volume *2*

Supplemental information

Reference-based comparison of adaptive immune receptor repertoires

Cédric R. Weber, Teresa Rubio, Longlong Wang, Wei Zhang, Philippe A. Robert, Rahmad Akbar, Igor Snapkov, Jinghua Wu, Marieke L. Kuijjer, Sonia Tarazona, Ana Conesa, Geir K. Sandve, Xiao Liu, Sai T. Reddy, and Victor Greiff

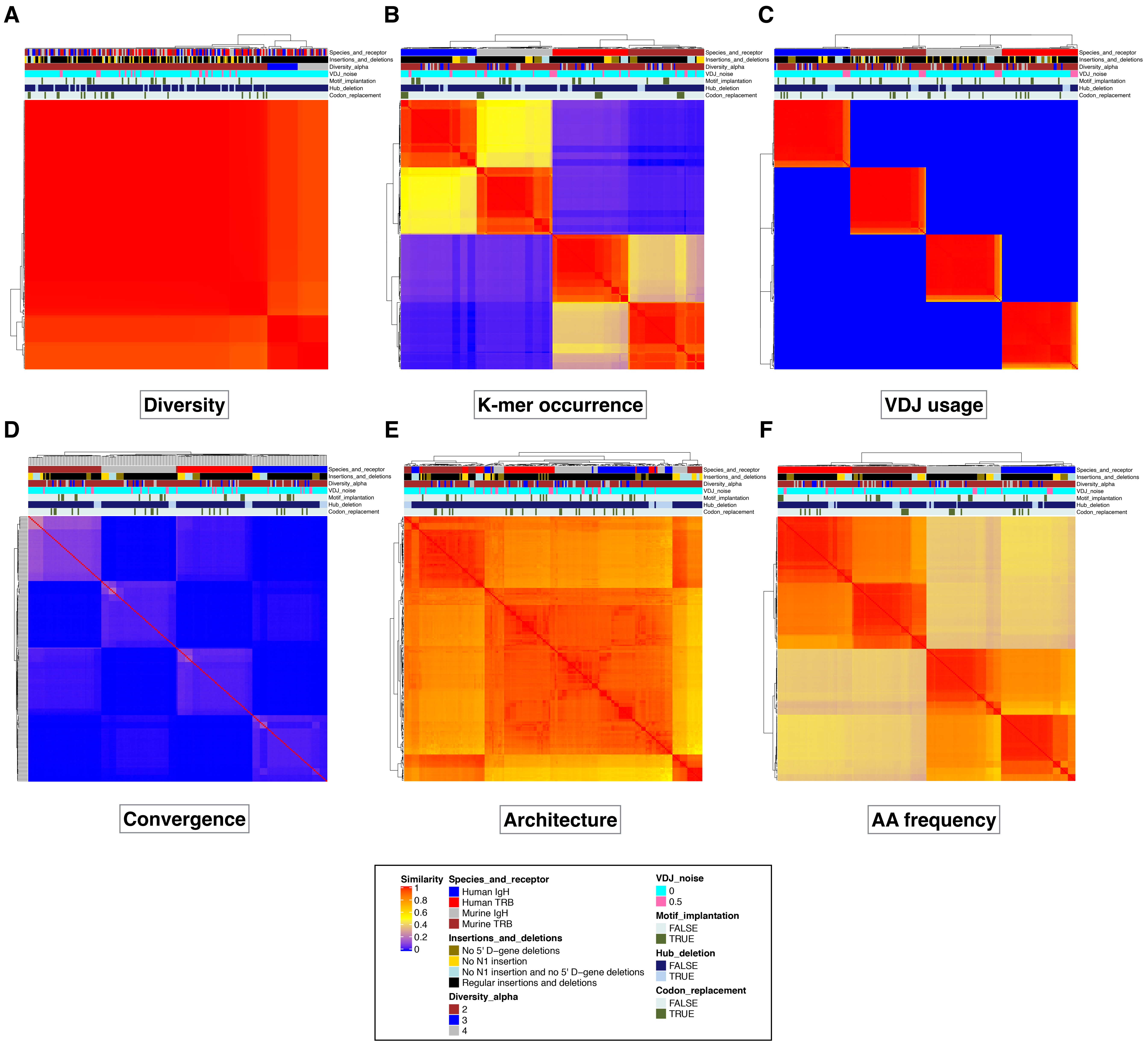

Supplementary Figure 1: Each single feature of the similarity network shows a unique topology, Related to Figure 2. (A–F) Heatmaps represent the similarity score landscape for each of the six features.

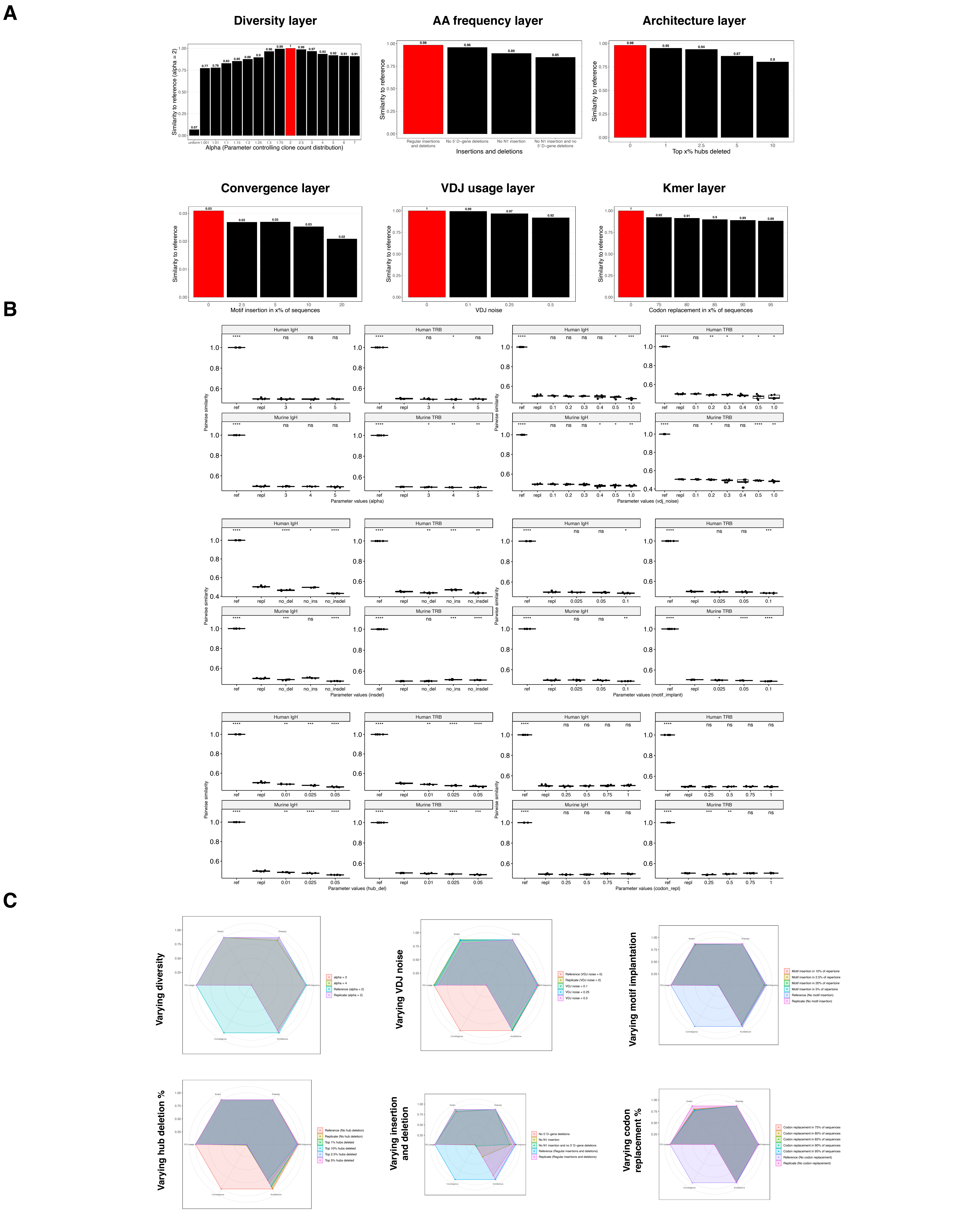

Supplementary Figure 2 I The similarity landscape of simulated repertoires defines a reference map for immune repertoire similarity. Related to Figure 2. (A) Per feature differences for a range of immuneSIM parameters (red bar indicates default parameters) (ns: p > 0.05, *: p <= 0.05, **: p <= 0.01, ***: p <= 0.001, ****: p <= 0.0001) **(B)** Similarities in each feature across different parameters (see also Fig 3A) (**(C)** Sensitivity of condensed similarity scores (y-axis) in response to different parameters (x-axis) faceted by species/receptor combination.

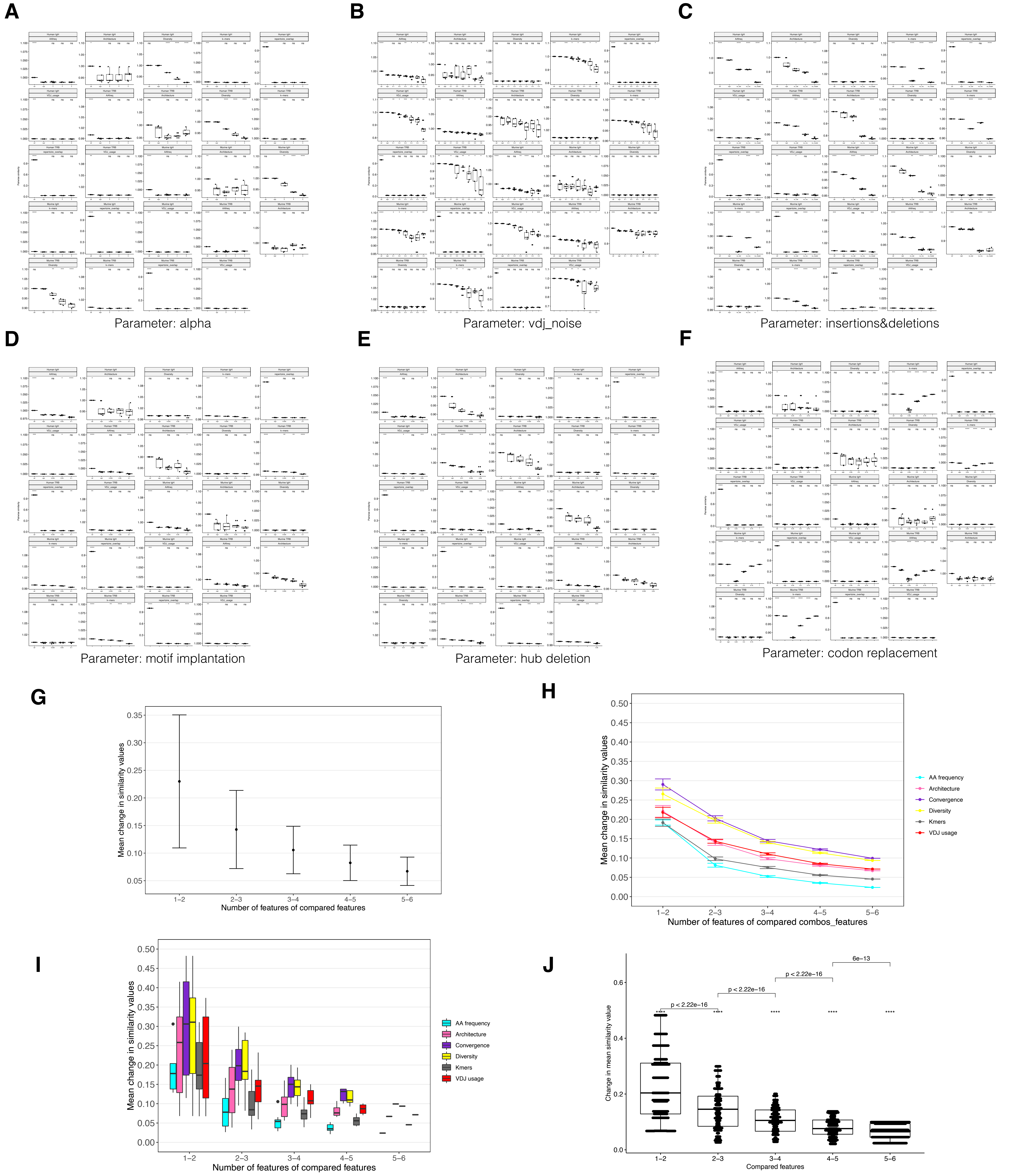

Supplementary Figure 3 I Sensitivity analysis per feature demonstrates the impact of each parameter change is feature specific and diminishing marginal utility of additional features Related to Figure 2. (A–F) The impact on pairwise similarity scores (y-axis) of parameter changes (x-axis) is shown for each parameter. Faceted by species/receptor combination and feature. **(G)** The mean change in similarity values after the addition of the next similarity feature (feature to be added is chosen randomly, 500 iterations) **(H)** The mean change in similarity values for the addition of each feature during various stages of the construction of the multi-feature network. **(I)** Boxplots of mean change in similarity values for the addition of each feature during various stages of constructing a multi-feature network. **(J)** Beeswarm representation of Fig. 2D. Error bars show mean ± standard deviation across iterations. Significance values are defined as ns: p > 0.05, *: p <= 0.05, **: p <= 0.01, ***: p <= 0.001, ****: p <= 0.0001.

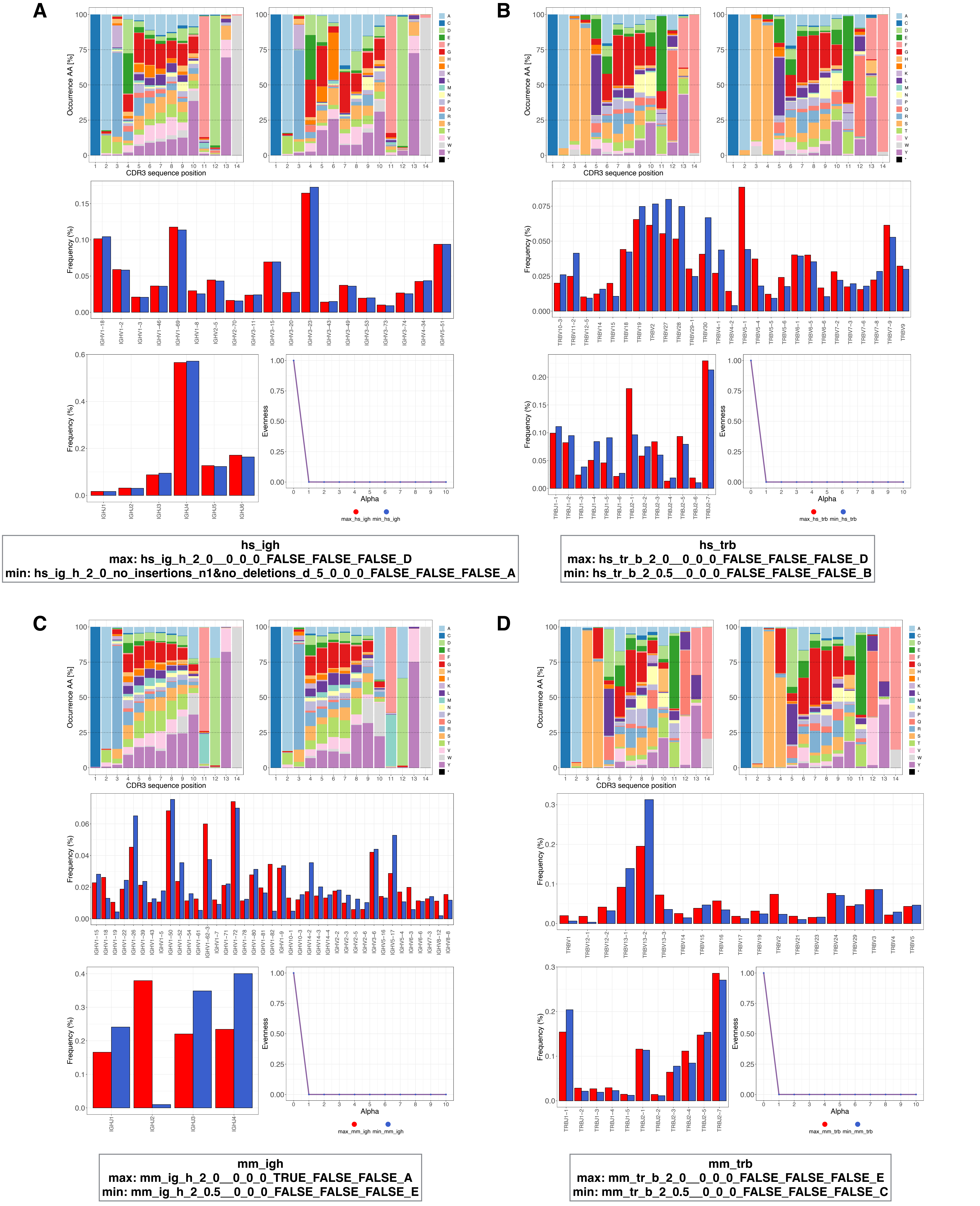

Supplementary Figure 4 | Within-cohort variability of simulated repertoires varies by receptor cohort (human and murine simulated repertoires). Related to Figure 3. The amino acid frequency (first row), VJ usage (middle row, bottom left), and evenness (bottom right) are compared between the most and least locally similar repertoires of human (hs) **(A)** IgH and **(B)** TRB simulated repertoires as well as mouse (mm) **(C)** IgH and **(D)** TRB simulated repertoires.

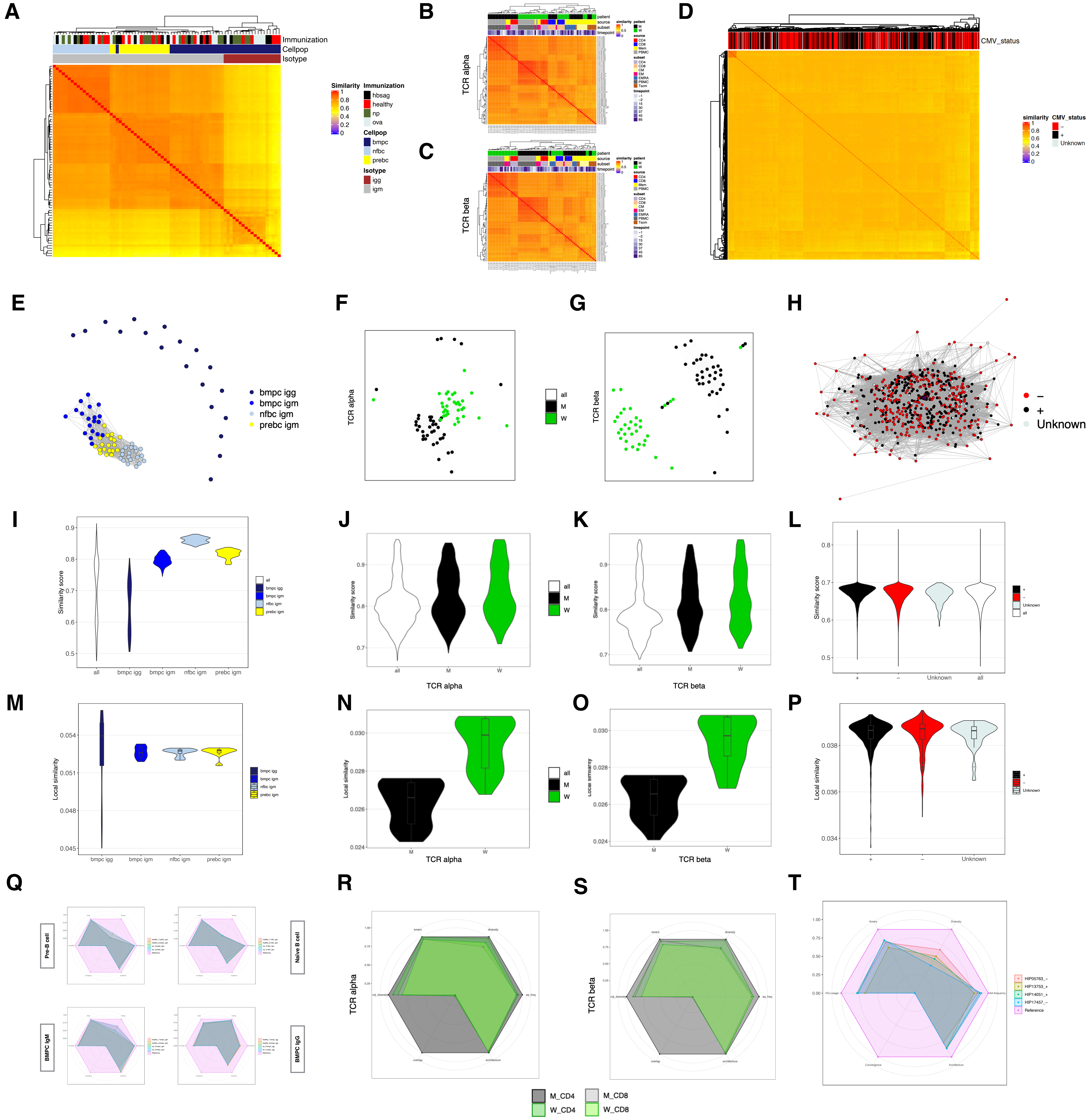

Supplementary Figure 5 | Application of immuneREF to 76 experimental repertoires from mouse immunization study (Greiff et al. 2017a), 72 T-cell repertoires from various cell populations of two Covid patients (longitudinal) and 666 experimental, CMV-serotyped repertoires (human, TCR, Emerson et al., 2017) finds even similarity distribution across repertoires of CMV+ and CMV- status. Related to Figure 4. (A) Similarity landscape of experimental (murine, BCR) repertoires from four immunization cohorts (Healthy, HBsAg, NP, OVA) and four cell populations (pre-B cell IgM, naïve B-cell IgM, bone marrow plasma cell IgM and IgG), **(B,C)** experimental (human, TCR, alpha and beta) repertoires for four cell populations **(D)** experimental (human, TCR) repertoires of CMV+ (289), CMV- (352) and unknown serotype (25) **(E–H)** Network visualizations of repertoires (nodes) and weighted edges between them representing similarity scores (top 25% edge by edge weights). **(I–L)** Distribution of similarity scores across the entire network and per cell population/condition shows variation within and across cohort. **(M–P)** Distribution of local similarity values per repertoire for each cell population/condition. **(Q–T)** Comparison of most locally similar repertoires of the conditions per cell population and immuneSIM reference repertoires (Reference, standard immuneSIM parameters).

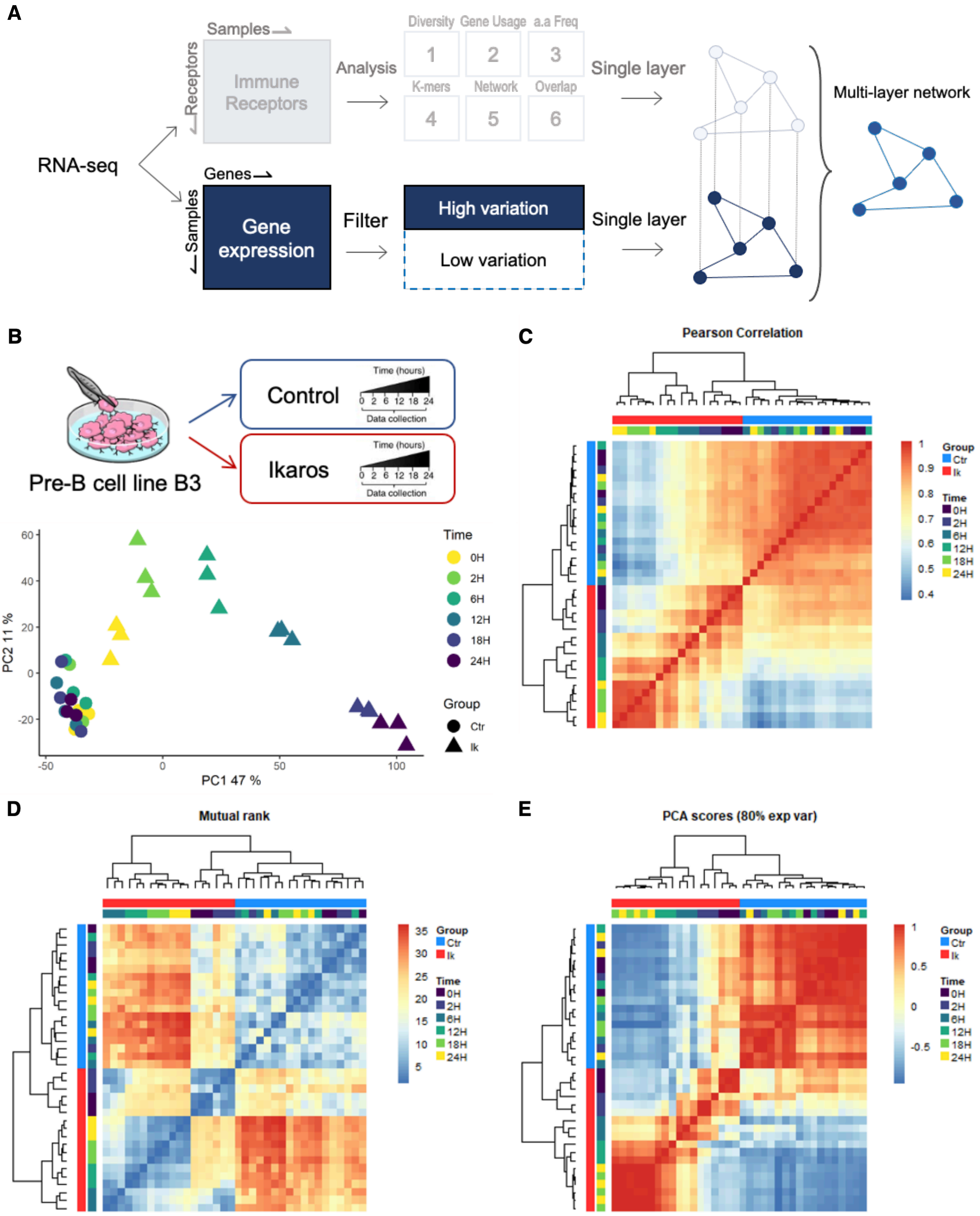

Supplementary Figure 6 I Gene expression and immune repertoire integration. Related to Figure 4. Gene expression can be added as an additional single feature to obtain the multi-feature network. **(A)** Pipeline overview. **(B)** Example of analysis using STATegra dataset (mouse pre-B cell line B3, RNA-seq, Gomez-Cabrero et al. 2019). **(C–E)** Heatmap representing co-expression patterns between samples using three methods available in the immuneREF package (Pearson Correlation, Mutual rank and PCA score).

Supplementary Table 1. Parameters for simulated datasets & hypothesized impact

**Parameter**	**Values/range**	**Hypothesized Impact (Feature)**
Alpha (clone count distribution)	**2**, 3, 4	Diversity feature only
Species	Human (hs), Mouse (mm)	All except diversity
Receptor chain	IgH, TRB	All except diversity
VDJ noise (introduces noise into germline gene frequencies)	**0**, 0.5	Germline gene usage, kmer, A.A. frequency, Architecture
Insertion Deletion Dropout (modifies inclusion of insertion and deletions)	**ins_and_del**, no_ins_n1, no_del_d_5, no_insertions_n1&no_deletions_d_5	Germline gene usage, kmer, A.A. frequency, Architecture
Motif implantation	True (implant "YAY" and "YVY" at a random position in 2.5% of sequences each), **False**	Convergence, (minor: architecture)
Hub deletion	True (Top 0.5 %), **False**	Architecture, Convergence
Synonym codon introduction	True ("tac" for "tat", "ggg" for "ggt" and "agc" for "agt", in 50% of sequences), **False**	K-mer, (minor: Convergence)

Supplementary Table 1 I ImmuneSIM parameters for simulated datasets & hypothesized impact. Related to Figure 2. The repertoires were simulated to differ across eight parameters. (In bold: Default parameters.) Please refer to immuneSIM documentation (https://immunesim.readthedocs.io/en/latest/parameters.html) for parameter definitions.

Supplementary Table 2. Quantitative description of high-throughput sequencing datasets.		
**Data Origin**	**Cell Type**	**No. of unique CDR3 sequences (mean±std.error)**
Mouse (C57BL/6J), B cell (Greiff et al., 2017a)	preBC (IGM) (19)<br>nBC (IGM) (19)<br>PC (IGM) (19)<br>PC (IGG) (19)	172’262 ± 9’394<br>395’558 ± 18’341<br>28’762 ±11’443<br>147 ± 18
Human (CMV-dataset), T cell (Emerson et al., 2017)	PBMC (CMV positive) (289)<br>PBMC (CMV negative) (351)<br>PBMC (CMV unknown) (26)	177’095 ± 4’235<br>186’951 ± 4’029<br>154’478 ± 14386
Human (Covid-dataset), T cell (TCRbeta) (Minervina et al., 2021)	CD4 (mild Covid) (10)<br>CD8 (mild Covid) (10)<br>Mem (mild Covid) (28)<br>PBMC (mild Covid) (24)	321’827 ± 34’100<br>189’289 ± 29’240<br>36’233 ± 6’232<br>623’003 ± 46’519
Human (Covid-dataset), T cell (TCRalpha) (Minervina et al., 2021)	CD4 (mild Covid) (10)<br>CD8 (mild Covid) (10)<br>Mem (mild Covid) (28)<br>PBMC (mild Covid) (24)	233’948 ± 32’085<br>141’119 ± 20’064<br>29’073 ± 5’253<br>425’560 ± 28’554
Human (BGI), T cell (Zhang et al., 2019)	PBMC (Healthy) (439))<br>PBMC (RA) (206)<br>PBMC (SLE) (877)	74’195 ± 25’364<br>123’292 ± 68’630<br>84’582 ± 52’944

Supplementary Table 2 I Quantitative description of high-throughput sequencing datasets. Related to Figure 4. Overview of the number of unique CDR3 sequences of the experimental datasets analyzed using immuneREF. Where the number of clonal sequences exceeded 10’000, the immune repertoire was subsampled by extracting the 10’000 clonal sequences with the highest clone count.
