# Supplementary material for: Reference-based comparison of adaptive immune receptor repertoires
Source: Cell Rep Methods. 2022 Aug 22;2(8):100269. doi: 10.1016/j.crmeth.2022.100269 (PMC9421535; doi:10.1016/j.crmeth.2022.100269)
Supplement: Document S2. Article plus supplemental information [file mmc2.pdf]

Cell Reports Methods

Article

Reference-based comparison of adaptive immune receptor repertoires

Graphical abstract

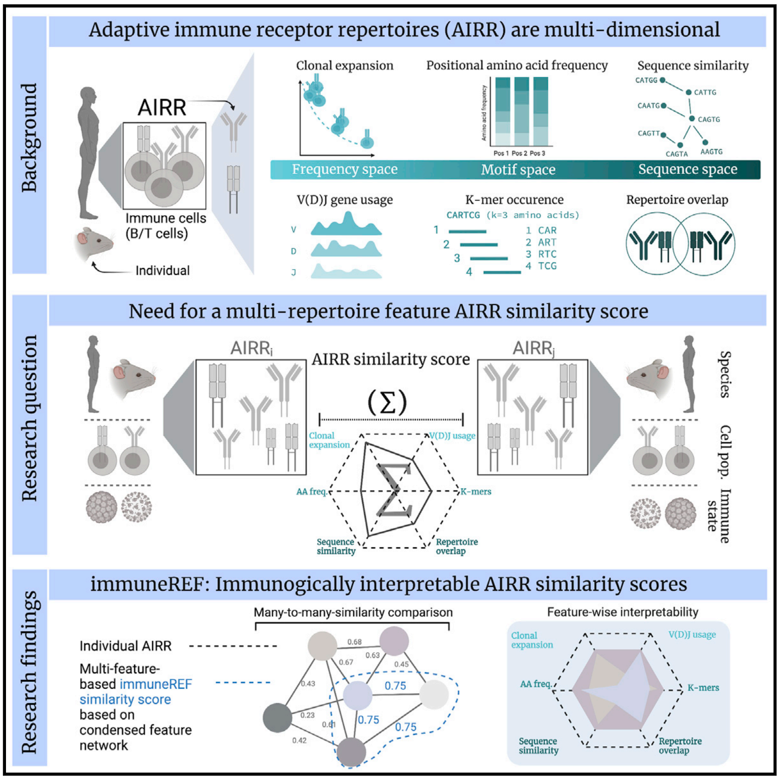

Highlights

- immuneREF enables reference-based comparison of immune repertoires
- immuneREF measures immune repertoire similarity with high sensitivity
- Only small similarity-based repertoire differences between health and autoimmunity
- Integration of gene expression with immune repertoire data

Authors

Cédric R. Weber, Teresa Rubio, Longlong Wang, ..., Xiao Liu, Sai T. Reddy, Victor Greiff

Correspondence

victor.greiff@medisin.uio.no

In brief

Weber et al. describe a method for reference-based comparison of adaptive immune receptor repertoires (immuneREF). immuneREF implements population-wide analysis of immune repertoire similarity, enabling the study of the adaptive immune response across health and disease states.

Weber et al., 2022, Cell Reports Methods *2*, 100269
August 22, 2022 © 2022 The Author(s).
https://doi.org/10.1016/j.crmeth.2022.100269

CellPress

Cell Reports Methods

CellPress
OPEN ACCESS

Article

Reference-based comparison of adaptive immune receptor repertoires

Cédric R. Weber,[1] Teresa Rubio,[2] Longlong Wang,[3,4] Wei Zhang,[3,5] Philippe A. Robert,[6] Rahmad Akbar,[6] Igor Snapkov,[6] Jinghua Wu,[3] Marieke L. Kuijjer,[7,8,9] Sonia Tarazona,[10] Ana Conesa,[11] Geir K. Sandve,[12] Xiao Liu,[3,13,14] Sai T. Reddy,[1,14] and Victor Greiff[6,14,15,*]

[1]Department of Biosystems Science and Engineering, ETH Zürich, Basel, Switzerland
[2]Laboratory of Neurobiology, Centro Investigación Príncipe Felipe, Valencia, Spain
[3]BGI-Shenzhen, Shenzhen, China
[4]BGI-Education Center, University of Chinese Academy of Sciences, Shenzhen, China
[5]Department of Computer Science, City University of Hong Kong, Hong Kong, China
[6]Department of Immunology and Oslo University Hospital, University of Oslo, Oslo, Norway
[7]Centre for Molecular Medicine Norway, University of Oslo, Oslo, Norway
[8]Department of Pathology, Leiden University Medical Center, Leiden, the Netherlands
[9]Leiden Center for Computational Oncology, Leiden University Medical Center, Leiden, the Netherlands
[10]Departamento de Estadística e Investigación Operativa Aplicadas y Calidad, Universitat Politècnica de València, Valencia, Spain
[11]Institute for Integrative Systems Biology, Spanish National Research Council, Valencia, Spain
[12]Department of Informatics, University of Oslo, Oslo, Norway
[13]Shenzhen International Graduate School, Tsinghua University, Shenzhen, China
[14]Senior author
[15]Lead contact
*Correspondence: victor.greiff@medisin.uio.no
https://doi.org/10.1016/j.crmeth.2022.100269

MOTIVATION B and T cell repertoires record past and current immune states. Therefore, the majority of immune repertoire studies aim to measure the impact of the immune state on the immune repertoire because it is widely assumed that repertoires change as a function of the immune state. So far, a method to measure and immunologically interpret differences between immune repertoires has remained unavailable. We have addressed the methodological challenge of immune repertoire comparison by implementing a reference-based multidimensional repertoire similarity measure based on *in silico* and experimental immunologically interpretable ground truth.

SUMMARY

B and T cell receptor (immune) repertoires can represent an individual's immune history. While current repertoire analysis methods aim to discriminate between health and disease states, they are typically based on only a limited number of parameters. Here, we introduce immuneREF: a quantitative multidimensional measure of adaptive immune repertoire (and transcriptome) similarity that allows interpretation of immune repertoire variation by relying on both repertoire features and cross-referencing of simulated and experimental datasets. To quantify immune repertoire similarity landscapes across health and disease, we applied immuneREF to >2,400 datasets from individuals with varying immune states (healthy, [autoimmune] disease, and infection). We discovered, in contrast to the current paradigm, that blood-derived immune repertoires of healthy and diseased individuals are highly similar for certain immune states, suggesting that repertoire changes to immune perturbations are less pronounced than previously thought. In conclusion, immuneREF enables the population-wide study of adaptive immune response similarity across immune states.

INTRODUCTION

B and T cell receptor (BCR, TCR) repertoires (also called adaptive immune receptor repertoires, AIRR) are continually shaped throughout the lifetime of an individual in response to environmental and pathogenic exposure. As of yet, however, there exists only a limited quantitative conception of how immune receptor repertoires differ across individuals and cell populations (Brown et al., 2019; Miho et al., 2018; Raybould et al., 2021). This is primarily because a method for measuring inter-individual

This is an open access article under the CC BY license (http://creativecommons.org/licenses/by/4.0/).

(inter-repertoire) similarity is lacking, thus greatly impeding the understanding of how health and disease shape immune repertoires and how disease contributes to the deviation of an individual's baseline repertoire (Cobey et al., 2015). Although it is generally thought that infection or disease induces measurable repertoire changes (even on the antigen-specific agnostic level), this belief remains unproven and, in fact, is counter to current evidence finding, using statistical learning, that even in systemic infections such as cytomegalovirus (CMV) only a comparatively very small number of TCRs are infection associated (DeWitt et al., 2018; Emerson et al., 2017; Pavlović et al., 2021). As opposed to machine learning approaches that aim to detect the most differentiating factors (i.e., subsets of a repertoire) between, for example, two different immune states (Greiff et al., 2020; Pavlović et al., 2021; Pertseva et al., 2021; Shemesh et al., 2021; Widrich et al., 2020a, 2020b), we investigate here a method for quantitatively comparing any two repertoires in an unsupervised fashion. We thus seek to understand to what extent individuals differ with respect to their entire repertoire and not just class-associated subsets.

The need for comparing immune repertoires using a quantitative measure has recently been addressed by approaches based on single sequence-dependent and sequence-independent features, which vary in statistical dependency (mutual information) and immunological interpretability (Chiffelle et al., 2020; Miho et al., 2018; Olson et al., 2019). Sequence-dependent approaches range from the measurement of clonal overlap (Bolen et al., 2017; Greiff et al., 2015a; Miho et al., 2018; Rognes et al., 2022; Yaari and Kleinstein, 2015) to more sophisticated algorithms that identify disease-specific enrichment of sequence clusters by testing against VDJ recombination models (Pogorelyy et al., 2019) or similarity networks of control datasets (Pogorelyy and Shugay, 2019; Shugay et al., 2015). Sequence-independent approaches are mainly represented by entropy-based diversity indices (Alon et al., 2021; Greiff et al., 2015a; Kaplinsky and Arnaout, 2016; Strauli and Hernandez, 2016), which have lately been augmented with a correction for sequence similarity (Arora et al., 2018; Vujović et al., 2021). None of the currently available comparative methods, which are based on single repertoire features, however, represent an integrated multi-feature measure of immune repertoire similarity that takes into account the complexity of information encoded in the ensemble of the existing immune repertoire features (Gupta et al., 2015; Heiden et al., 2014; Nazarov et al., 2020; Shugay et al., 2015). Such an integrated measure, encoding per-feature similarity in one common mathematical structure, is needed to enable a representation of repertoire similarity.

Here, we introduce immuneREF: a measure for quantifying immune repertoire similarity across multiple immune repertoire features. Our framework, implemented in an R package, measures immune repertoire similarity using a combination of features that are immunologically interpretable (clonal expansion, sequence composition, repertoire architecture, and clonal overlap) and that cover largely distinct dimensions of the immune repertoire spaces. Specifically, to interpret immune repertoire similarity scores, immuneREF establishes a self-augmenting dictionary of simulated and experimental datasets where each new dataset analyzed may be used as a comparative reference for scoring and biologically interpreting inter-individual variation (and thus the deviation) of immune repertoire features (Figure 1). We applied immuneREF to >2,400 immune repertoires from humans with varying immune states (healthy, virus infection, autoimmune disease) and found that the similarity of blood-derived immune repertoires is not consistently a function of the immune state.

Overall, immuneREF enables the quantification of repertoire similarity at population scale while still providing single-individual resolution, and it enables answering fundamental questions such as to what extent immune repertoires are robust to perturbations introduced by immune events.

RESULTS

Reference-based comparison of immune repertoires based on immunological features: Constructing a similarity atlas of immune repertoires

To derive a similarity measure for immune repertoires, we devised a framework that calculates a repertoire similarity score based on six features that reflect immune repertoire biology (Figure 1). These features are (1) germline gene diversity (Greiff et al., 2015a; Yaari and Kleinstein, 2015), (2) clonal diversity (Greiff et al., 2015a; Stern et al., 2014), (3) clonal overlap (Greiff et al., 2015a; Yaari and Kleinstein, 2015), (4) positional amino acid frequencies (Mason et al., 2019), (5) repertoire similarity architecture (Bashford-Rogers et al., 2013; Ben-Hamo and Efroni, 2011; Miho et al., 2019), and (6) k-mer occurrence (Greiff et al., 2017b; Thomas et al., 2014) (see the STAR Methods section for a detailed immunological and mathematical description of these features). A similarity score is calculated for each pair of repertoires and each feature (six $n \times n$ symmetric matrices, n = number of repertoires), creating a similarity matrix for each feature. This matrix may be viewed as a weighted network, in which the nodes correspond to repertoires and the edges connecting the nodes are the similarity scores. The resulting six single-feature similarity networks enable insight into per-feature similarity. Finally, a composite network of the six feature similarity networks represents an interpretable multidimensional picture of the repertoire landscape. Briefly, the single features are condensed into a multi-feature composite network by taking the mean of all single-feature similarity values resulting in a single repertoire similarity value (for alternative approaches to computing composite networks, see STAR Methods section). By virtue of representing a similarity matrix as a weighted network repertoire, similarity may be computed on selected levels such as one (repertoire) to many (repertoires), many to one, and many to many (Figure 1). Interpretability stems from all repertoire features being transformed into a similarity measure on a 0–1 scale allowing for direct quantification of their individual contribution to multidimensional immune repertoire similarity.

immuneREF measures immune repertoire similarity with high sensitivity

We sought to quantify the sensitivity by which immuneREF can detect differences between immune repertoires with respect to the six repertoire features. The simulated repertoires, varying in a controlled manner, represent a ground truth reference map that enables a more precise assessment of immuneREF sensitivity. For example, simulated repertoires may be used to guide the evaluation of variation between experimental repertoires

CellPress
OPEN ACCESS

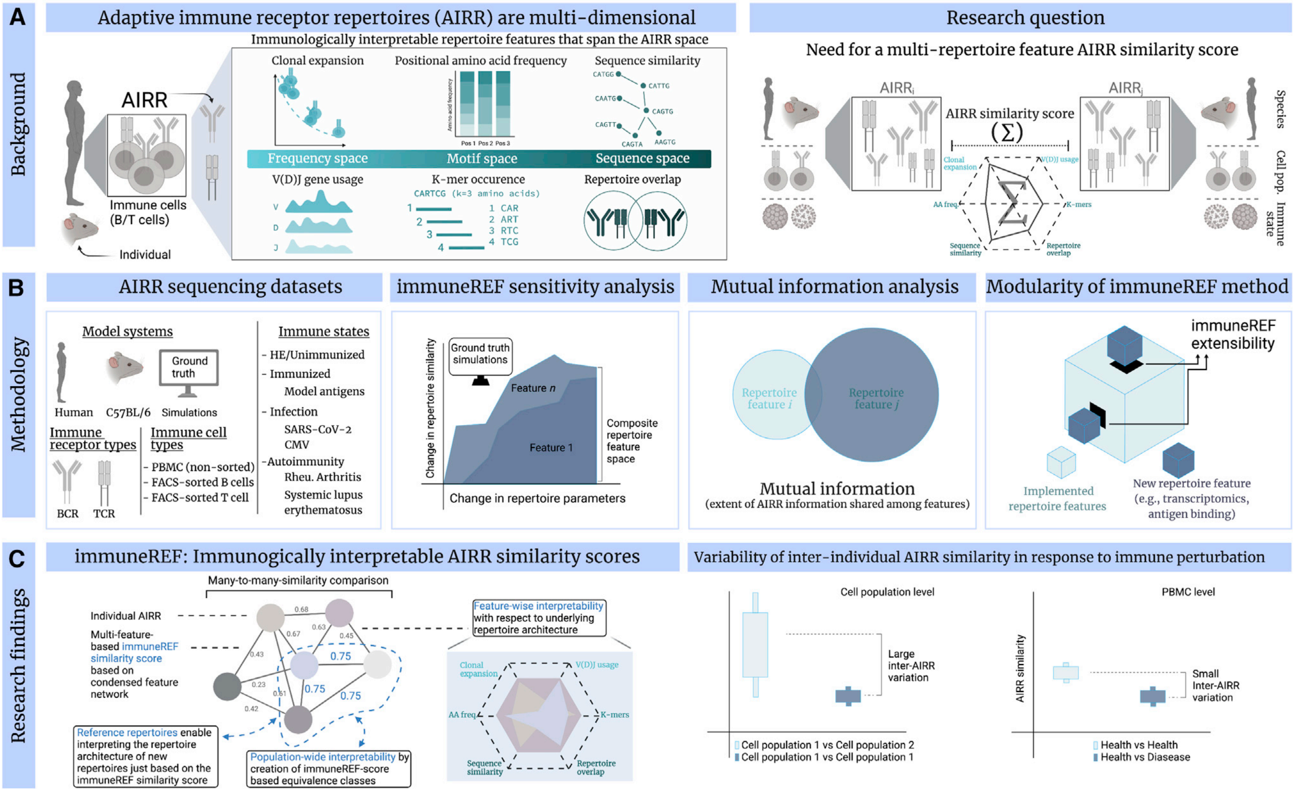

Figure 1. | Reference-based comparison of adaptive immune receptor repertoires (AIRRs)

(A) The complexity of AIRRs spans the frequency, motif, and feature space to each, of which distinct repertoire features may be attributed: the immune information stored in AIRRs is multidimensional. A longstanding question in the AIRR field is how to quantitatively measure inter-sample (sample, e.g., individual, immune cell population) AIRR similarity by accounting for AIRR feature multidimensionality in the effort to understand the distribution of inter-sample AIRR similarity across different immune events or immune cell populations.

(B) We set out to develop an AIRR similarity measure that is sensitive, captures maximal immune information, and is sufficiently flexible to allow future integration of additional repertoire features (extensibility).

(C) Each AIRR is represented as a node in a similarity network. The edges connecting the nodes represent the similarity score between the AIRR based on the six repertoire features. The immuneREF approach establishes interpretability on different levels: (1) from a single-feature perspective, the application of spider plots allows for an interpretable comparative analysis between repertoires, enabling the user to interpret the result observed in the condensed network on a per feature basis. (2) From the condensed feature network perspective, a major novelty introduced by the immuneREF workflow is the ability to combine established repertoire features into a common coordinate system. This transformation allows the combination of trends across features into a single condensed network that represents pairwise-cross-feature similarities. These pairwise similarities allow for the identification of subsets of more similar or aberrant repertoires. Interpretability on both features means allowing comparison to other repertoires and to simulated ones (of which we know the repertoire structure as ground truth), thus creating similarity equivalence classes. Equivalence classes create sets of reference repertoires, which enable interpreting the repertoire structures of other repertoires solely based on the immuneREF similarity score.

with respect to each repertoire feature as well as multi-feature combinations. Simulations were performed using the immuneSIM repertoire simulation suite (Weber et al., 2019), which was used to create native-like repertoires that were varied across eight parameters. Native-likeness was demonstrated in Weber et al. (2019). The parameters that were varied across simulated repertoires included clone count distribution, V-, (D-), J-gene frequency noise, insertion, and deletion likelihoods, species (human and mouse), and receptor type (IgH, TRB). We constructed additional simulated repertoires with spiked-in motifs (mimicking antigen-binding motifs; Akbar et al., 2019), excluded hub sequences in the sequence similarity network (simulating network architecture variation; Miho et al., 2019), and replaced nucleotide codons with synonymous codons (simulating biases in the k-mer occurrence that are relevant in detectable immunogenomic patterns of public clones; Greiff et al., 2017b) (see STAR Methods; Table S1 lists the parameter variations used for the simulations and how each of the parameters is expected to influence the six immuneREF features). The parameter combinations were chosen so each simulated repertoire varied only along one parameter dimension at a time, allowing us to determine the sensitivity of each feature to each parameter change.

The mathematical structure of the single-feature similarity matrices enables their merging into a composite network that provides the opportunity for a condensed single-score representation of inter-sample repertoire similarity. The composite immuneREF network (which combines all six repertoire features) recovers major variation in the repertoires including noise

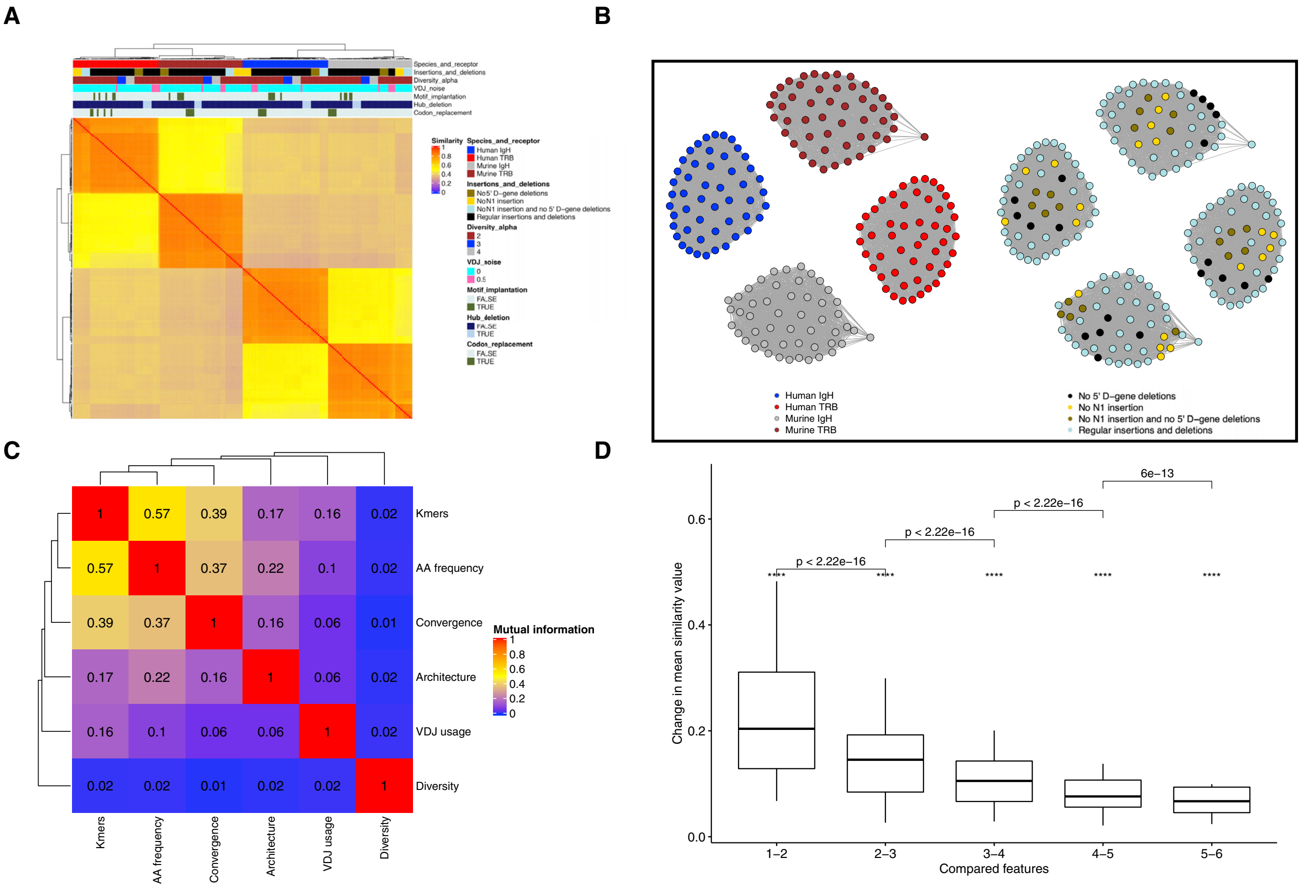

Figure 2. immuneREF measures immune repertoire similarity with high sensitivity using features that capture immune repertoire biology

We simulated 200 immune repertoires using 40 different parameter combinations (in quintuplicate).

(A) Hierarchical clustering visualizes the sensitivity of immuneREF by the successful grouping of immune repertoires that were simulated with slightly different parameters (composite network; see main text for details).

(B) Network visualization with simulated repertoires as nodes and weighted edges between repertoires of similarity values above the upper quartile.

(C) Quantification of mutual information among immune repertoire features.

(D) Change in mean similarity of composite networks of increasing number of features. t test significance values are defined as ns: $p \geq 0.05$, *: $p < 0.05$, **: $p \leq 0.01$, ***: $p \leq 0.001$, ****: $p \leq 0.0001$.

introducing parameter changes (Figures 2A and 2B). immuneREF also clearly distinguishes repertoires from different receptors and species based on strongly distinguishing features such as V-, (D-), and J-gene usage while allowing the identification of commonalities in amino acid usage, clonal diversity, and architecture across immune receptors and species. This sensitivity analysis also underlines a major advantage of immuneREF, namely its flexibility to accommodate both BCR and TCR repertoires from different species in one single analysis workflow.

We quantified the sensitivity of immuneREF by detecting significant changes in similarity scores corresponding to the variation in simulation parameters across both the single feature (Figures S1–S3) and composite network (Figure 2A) and found that each feature had a unique sensitivity profile to changes in the simulation parameters, underscoring the value of per-feature similarity evaluation. For example, a change in the alpha parameter of the Hill function (controlling clone count distribution) solely impacted the immuneREF diversity feature. As the immuneSIM parameter controlling the distribution of clone counts only affects the clone count simulation without impacting simulated sequences, the fact that only the feature targeted by the parameter change is impacted shows that immuneREF is robust to random noise in the simulation that is not introduced through parameter changes. An increase in the V(D)J noise parameter, which modifies the frequencies of the germline genes used in the simulation, led to detectable and significant changes in similarities of the germline gene usage and k-mer occurrence features. Modification of the insertion/deletion patterns (dropout of deletions and or insertions) led to a consistent impact in the amino acid frequency feature and, more importantly, the architecture feature, where a lower diversity due to restricted insertions and deletions led to significant changes in network architecture. Implanting motifs at various frequencies led to a significant similarity change in the k-mer occurrence feature. The deletion of hub sequences led to an impact in the architecture feature and also changed the repertoire overlap similarity,

thus underlining the importance of public clones in the network architecture as reported previously (Miho et al., 2019). Finally, we modified the repertoires by introducing synonymous codons at various percentages and found that the k-mer occurrence feature was the only one impacted. Therefore, we conclude that immuneREF features largely react as hypothesized to variation in simulation parameters (Table S1). Taken together, we demonstrated that the immuneREF framework is sensitive to even comparatively small repertoire variations.

Mutual information analysis demonstrates no inter-dependence to limited inter-dependence of immuneREF features

While the examined features were initially chosen based on immunological criteria, we also wished to verify whether each feature provides a sufficiently different measurement of the immune repertoire information space (Figure 2C). Specifically, having integrated all features into a common coordinate system, we were able to compute cross-feature mutual information and found that features show no dependence to limited dependence (range = 0.01–0.57; Figure 2C) indicating largely non-overlapping and distinct spaces of immune information captured. The highest mutual information was found between the positional and sequential sequence-derived features (i.e., positional amino acid frequency and gapped k-mer occurrence, respectively), whereas the lowest mutual information value was found between the diversity and convergence features (Figure 2C).

Complementarily, we sought to quantify to what extent the addition of new repertoire features leads to diminishing returns (sufficiency analysis). To this end, we computed the mean change in repertoire similarity values when increasing the number of features from one through six. Thereby, we could show that each additional feature added increasingly less information, as shown by the diminishing change of the mean similarity value with each added feature. The saturation of the mean similarity change curve indicated information saturation independent of the order in which features were arranged (Figures 2D and S3G–S3J). As discussed below, mutual information values behaved similarly for experimental repertoire data. Thus, we demonstrated that the immuneREF framework creates information-laden similarity networks, whose topologies capture the immunological similarity landscape of immune repertoires.

The similarity landscape of simulated repertoires defines reference repertoires

By calculating the similarity matrix for each of the six immune repertoire features, we embedded the six different immunological features into a common coordinate system, i.e., a network structure. This network (with nodes representing repertoires and weighted edges representing pairwise similarity) situates each repertoire within a similarity landscape allowing quantification of many-to-many repertoire similarity.

A more fine-grained image of the similarity landscape may be gained by examining the similarity from the perspective of every single repertoire (Figures 3C and 3D). We define the local similarity of a repertoire to its neighboring repertoires as a scaled node strength (see STAR Methods). This local similarity represents the position of the repertoire with respect to its direct neighbors in its cohort (defined by an application-dependent label, e.g., same species and disease) and allows us to distinguish between well embedded and aberrant repertoires. The local similarity measure further acts as a magnifying glass by elucidating finer differences between repertoires, which are diluted by population averages when examining repertoire similarity across the full similarity network. Using this perspective, repertoires that are most (locally) similar to other repertoires in their cohort can be identified, allowing the extraction of repertoires most representative for a given immune state. Such detailed one-to-one feature comparisons highlight, in the most simple case, which features of the simulated repertoires are receptor specific (amino acid frequency, k-mer occurrence, VDJ usage, and convergence) and which are more general to immune repertoire data showing higher similarity across different species and receptors (diversity, architecture) (Figures 3E and S4).

Having evaluated the similarity of simulated datasets, these may serve as a reference to interpreting similarity score variation of experimental repertoires (Figure 3C), thus enabling the creation of equivalence classes of immune repertoires not only as previously performed based on clonal expansion (Greiff et al., 2015b) but based on six repertoire features. Furthermore, any evaluated repertoire, be it of experimental or simulation origin, will become a new node in the similarity network and may serve as a valid reference point (just as any other node in the network). This network of self-augmenting repertoire similarity reference points is another source of interpretability as it allows the linking of the repertoire similarity of any number of repertoires with their underlying features. In the next section, we provide such a repertoire similarity network on experimental datasets.

Validation of immuneREF on experimental data: Detection of differences between cell populations in mouse immunization and human COVID-19 datasets

To validate immuneREF sensitivity on experimental data, we used antibody repertoire datasets generated from a mouse antigen immunization study, where differences in the similarity between antigen immunization cohorts are expected (Greiff et al., 2017a, 2017b; Miho et al., 2019). Notably, we were able to recover clear differences between isotypes and cell populations (both with higher within-cohort and lower across-cohort similarity); additionally, we found that the antigen immunization cohorts have more distinct similarity profiles in the plasma cell populations (IgG) compared with the antigen-inexperienced cell populations (Figure S5). The overall high similarity scores across the full immunological feature range are in agreement with our previous studies where we observed high similarity between these repertoires on a single feature basis (Greiff et al., 2017a).

Similarly, applying immuneREF to TCR repertoires of patients recovered from mild cases of COVID-19 (Minervina et al., 2021) revealed clusters of increased similarity within patients and cell populations (Figure S5).

Application of immuneREF to >1,500 experimental blood immune repertoires indicates only small similarity-based differences between health and autoimmune disease

Having established the sensitivity of our approach in detecting a wide range of differences between simulated repertoires

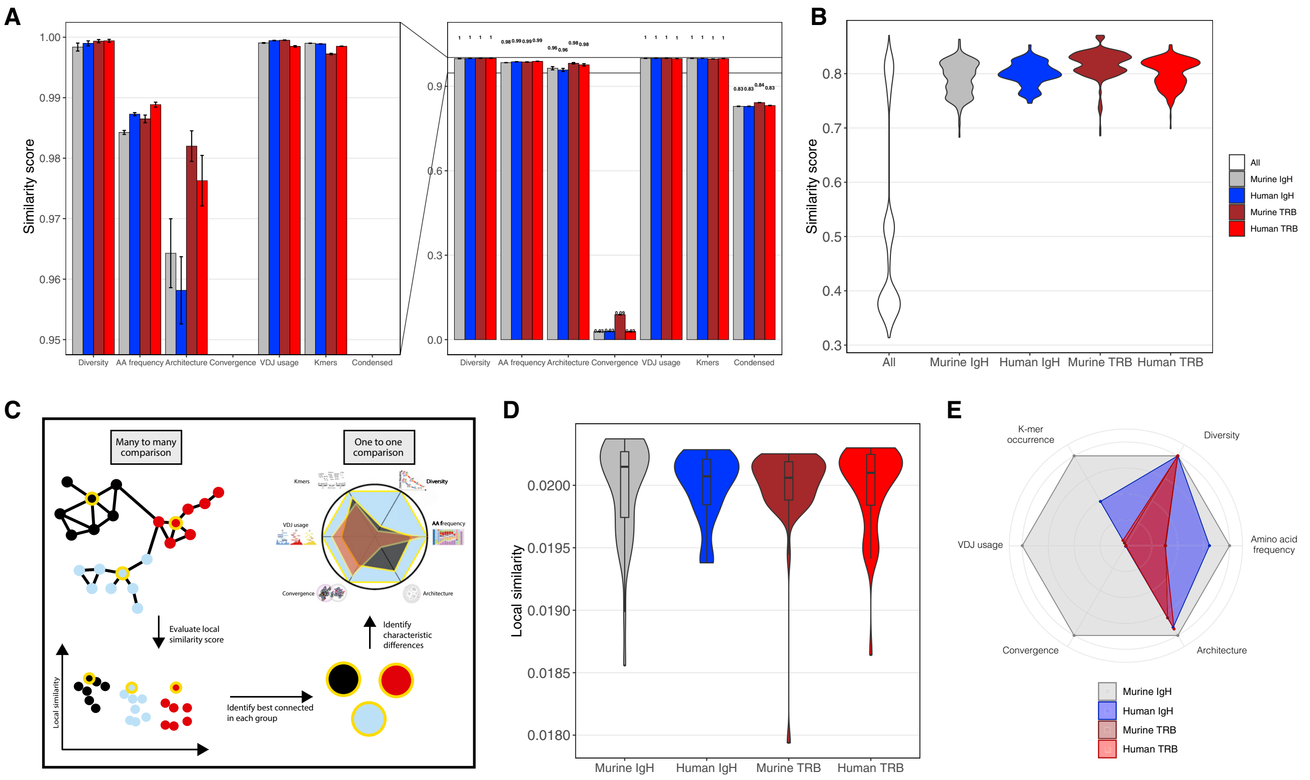

Figure 3. The similarity landscape of simulated repertoires defines reference repertoires

(A) Baseline similarity between replicates for repertoires simulated using default immuneSIM parameters (see Table S1) is $\geq$0.96 for five of six features, with the convergence feature being the exception by definition at $\leq$0.09. Bar graphs show mean SEM across replicates.

(B) Repertoire similarity distribution in a condensed network across the various evaluated parameter range. Across cohorts, similarity scores have a broad range, whereas within cohorts the range is more restricted.

(C) Workflow to determine representative repertoires per cohort going from many-to-many to a one-to-one comparison.

(D) Local similarity distribution per species/receptor combination enables situating each repertoire based on its connectivity with respect to neighbors in the same cohort.

(E) Comparing repertoires with maximal local similarity in their cohort visualizes the commonalities between receptor types; here the Murine IgH repertoire with maximal local similarity serves as a reference repertoire. The plot visualizes the similarities of each non-reference repertoire to the Murine IgH reference.

(Figures 2 and 3) and between experimental repertoires of different B cell populations (Figure S5) with respect to immunologically relevant and interpretable repertoire features, we set out to determine the similarity landscape of large-scale experimental TCR repertoire datasets. We evaluated 1,522 human TCR repertoires derived from peripheral blood mononuclear cells (PBMCs) of patients with varying and diverse immune states (PanImmune Repertoire Database (PIRD) dataset containing samples from healthy, rheumatoid arthritis (RA), and systemic lupus erythematosus (SLE) patients; Table S2). We found an even similarity landscape of overall high similarity scores (Figure 4A). Similarity score distribution was also even in single features, which despite feature-specific differences, show overall high similarity scores between repertoires. We examined networks at three different similarity cutoffs (an edge is drawn between two repertoire nodes if their similarity is in 25%, 50%, and 75% top weights, respectively), and we found that in all three cases, no immune state-specific grouping could be observed (Figure 4B).

The range of general and local similarities across all samples as well as within each disease cohort was evaluated using an analogous approach to that used for the simulated datasets (Figures 4C and 4D). While the similarity scores ranged between ~0.5 and 0.8 overall, the within-disease cohort spread varied, with the healthy and RA cohorts showing a more restricted range of similarity scores compared with a broader range for SLE (Figures 4C and 4D).

To quantify per feature similarity and dissimilarity with respect to a reference dataset, we compared the repertoires identified as the ones best connected (highest local similarity) within their cohort to an immuneSIM reference repertoire (human, TRB, standard parameters; see STAR Methods) (Figure 4E). The similarity scores of all tested immune states largely overlap with respect to the healthy reference repertoire, with convergence being the feature dimension with the largest dissimilarity, meaning there is almost no convergence between the RA or SLE samples and the reference.

Following our observations of high repertoire similarity within the PIRD dataset, we ran immuneREF on another large publicly available dataset (human, TCR) (Emerson et al., 2017) with yet another difference in immune state (CMV). The dataset contains

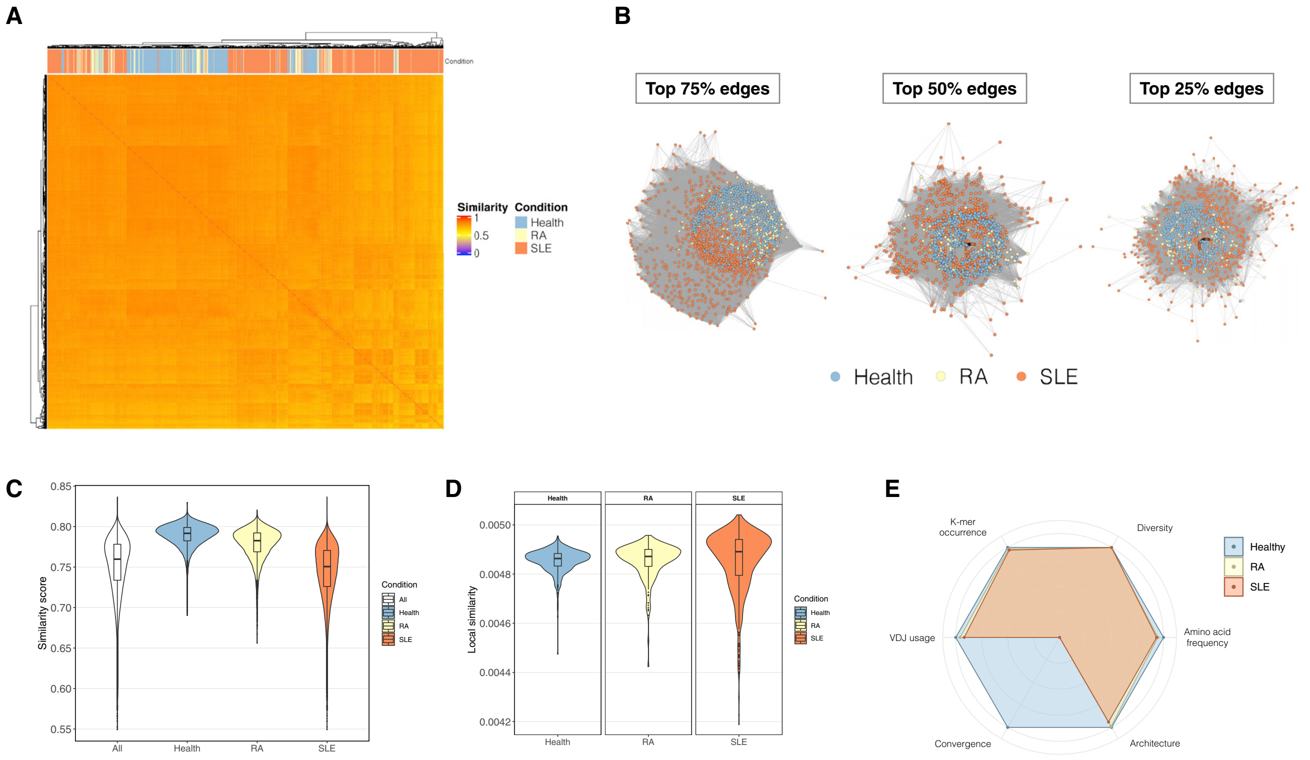

Figure 4. Application of immuneREF to 1,522 experimental repertoires

(A) Similarity landscape of experimental (human, TCR) repertoires across three immune states (healthy, 439 repertoires; rheumatoid arthritis, 206 repertoires; and systemic lupus erythematosus, 877 repertoires).

(B) Network visualization of the 1,721 nodes and weighted edges between repertoires of similarity scores (at three cutoff levels, 25%, 50%, and 75% top edge weights).

(C) Distribution of similarity scores across the entire network and per immune state shows different degrees of within-cohort homogeneity.

(D) Distribution of local similarity values per repertoire, faceted by cohort.

(E) Comparison of the repertoires with the highest local similarity per immune state and an immuneSIM reference repertoire (default immuneSIM parameters; see Table S1).

666 PBMC samples of which 289 are from CMV-positive patients, 351 are from CMV-negative patients, and 26 are from patients with unknown CMV status. This dataset has previously been used to showcase immune state classification with high accuracy via the identification of CMV-associated public TCR sequences (sequences shared between individuals). In a similar fashion, immune state-associated public sequences were used to successfully classify RA and SLE samples from the PIRD dataset (Liu et al., 2019). As with the PIRD dataset, we observed high within and across immune state repertoire similarity (Figure S5). This is in line with the findings of Emerson and colleagues as they found that only a small subset of clones (CMV-associated ones in Emerson et al., 2017) significantly differed in abundance between immune states (CMV+, CMV–) and that that shared antigen exposure to CMV led to a reduced number of shared TCRβ clones, even after controlling for individual human leukocyte antigen (HLA) type, indicating a largely private response to a major viral antigenic exposure (Johnson et al., 2021).

In summary, the results of our analysis of human TCR repertoires strongly support the argument that the signal-to-noise ratios, where signal means repertoire features associated with disease status, are unfavorably tilted toward noise, where noise is defined as technological and immunological information, which cannot *yet* be linked to a given disease state.

Extensibility of immuneREF: Integration of gene expression with immune repertoire data

The mathematical structure of the composite network obtained from immuneREF allows the extensibility of the immuneREF framework to other features. As proof of principle of this immuneREF capability, we show here an integrative analysis of immune repertoires and gene expression. This integration is of high interest to RNA-seq experiments that include both receptor and global transcript sequences, or even repertoire experiments paired with transcriptomics (Rubio et al., 2022; Song et al., 2021). Integration of immune repertoire with gene expression is challenging due to the multidimensional nature of both kinds of datasets and the discrepancy in their data structure. Previous attempts of integration are still over-simplistic, such as the calculation of correlation between the number of distinct CDR3 amino acid sequences and gene expression of some marker genes such as *CD3*, *CD4*, *CD8*, HLA class I, and class II genes (Brown et al., 2015).

immuneREF includes the option to evaluate similarity based on a gene expression matrix and add it to the composite

network. Briefly, immuneREF first filters all genes with low variation between experimental conditions and then calculates the pairwise correlation between observations to construct a single gene expression feature (similarity matrix). Once the seven features (six from immune repertoires and one for gene expression) are calculated, they may be condensed into a multi-feature network as described above. Our solution for integrating receptors with gene expression confers immuneREF the advantage of overlaying dual biological information (Figure S6A).

As an example, we analyzed bulk RNA-seq gene expression of pre-B cell line B3 from the published STATegra project (Gomez-Cabrero et al., 2019). This is a time-course experiment that collects samples at six time points using an inducible Ikaros system where B cell progenitors undergo growth arrest and differentiation (Figure S6B). Principal-component analysis (PCA) showed clear differences at gene expression level when control and Ikaros groups were compared but also within the Ikaros group across time, being t0 the nearest to controls (Figure S6B). To generate the single-feature similarity matrix of gene expression that better collects these differences, we tested the three available correlation-based methods implemented in immuneREF (Figures S6C–S6E). All of them perfectly separated control (blue) and Ikaros (red) groups. Additionally, ‘‘Pearson correlation’’ and ‘‘PCA scores’’ nearly recovered correctly the time series pattern (purple to yellow degradation), while mutual rank matched perfectly.

DISCUSSION

Combining methods from both immune repertoire and network analysis, we have provided a framework for flexible reference-based quantification of immune repertoire similarity. Using ground truth simulations, we show that immuneREF is sensitive to inter-repertoire differences in all immunological features. Taking advantage of information theory, we showed using both simulated and experimental data that the features selected for immuneREF cover a large extent of immune repertoire biology. We introduced the concepts of full-network repertoire similarity and local similarity, which allow complementary quantification of the impact of the differences in the repertoire similarity landscape. Specifically, while the more general repertoire similarity evaluated on the entire network provides insight into the range of similarity within and across conditions, local similarity shows a particular advantage of the network approach, as the embedding of a repertoire in its neighborhood can markedly differ from what can be expected by its pairwise connections.

immuneREF not only provides a framework for measuring immune repertoire similarity but also for interpreting it. Specifically, it enables the creation of equivalence classes of immune repertoires lacking from existing methods. For example, once the similarity observed within a given set of experimentally obtained immune repertoires has been computed, such repertoires may function as reference points that in turn enable the interpretation of relative similarity in other repertoires (Figures 1 and 3C). Of note, the concept of diversity measures creating equivalence classes has been noted previously for Hill diversity measures (Greiff et al., 2015b) and is here extended to include additional repertoire features immuneREF unifies as single and composite features, frequency-dependent, and sequence-dependent similarity measures into one computational framework. Beyond quantifying the repertoire similarity of experimental immune repertoires, immuneREF also enables the comparison of simulated (Han et al., 2021; Marcou et al., 2017; Safonova et al., 2015; Weber et al., 2020; Marcou et al., 2017; Safonova et al., 2015; Weber et al., 2019) and *in vitro* synthetic immune repertoires used for therapeutic antibody discovery (Mason et al., 2018). Furthermore, immuneREF may be used for data curation purposes in immune repertoire databases such as iReceptor (Corrie et al., 2018), VDJserver (Cowell et al., 2015), PIRD (Zhang et al., 2019), and Observed Antibody Space (Kovaltsuk et al., 2018). Specifically, upon the integration of an immune repertoire into a database, the similarity of the repertoire with all other stored repertoires may be computed. Beyond immunological insight, immuneREF may reveal unexpected technological variation, thus motivating follow-up inspection (Barennes et al., 2021). Since immuneREF has been built to work across species, cell populations, and receptor types and experimental or simulated data (all-in-one comparative framework), it enables rapid distinction of cohort-specific and cohort-unspecific features. This is also important for comparative immunological approaches not centered on health versus disease comparison but, for example, the evolution of adaptive immunity (Pancer and Cooper, 2006).

The ease of use of the immuneREF approach opens new possibilities for large-scale comparative studies as shown on the PIRD dataset, which may yield additional insight into the challenges of predicting immune state based on repertoire profiling. Indeed, we found that the population average quantified by immuneREF may ’’conceal’’ relevant immunological phenotype signals, despite the fact that the sensitivity of immuneREF was shown to be high in simulated and experimental data (Figures 2 and S5). Given the lack of large-scale (antigen-specific) data, it remains unclear how the information of the immune state is distributed across immunological features. Specifically, our finding—that repertoire similarity does not differ across immune states—is strictly only valid for unsorted PBMC TCR repertoire data as examined in this study. As known from previous studies (Amoriello et al., 2020, 2021; Csepregi et al., 2021; Ghraichy et al., 2021; Greiff et al., 2017b; Li et al., 2020; Ota et al., 2022; Riedel et al., 2020; Rosati et al., 2021), different cell populations (in different lymphoid organs) may behave in a highly different manner (Figure S5). On the other hand, it did not escape our attention that this broad similarity in human blood samples might suggest the maintenance of lymphocyte homeostasis even in the event of chronic disease.

Our results reinforce the notion that while some diseases may introduce abnormalities into the immune repertoire, others result in a comparatively normal one (Bashford-Rogers et al., 2019), a result that suggests the absence of a signature unique to health. If this is true, then blood-based immune repertoire diagnostics will require even more advanced methodologies to be developed (Arnaout et al., 2021; Dahal-Koirala et al., 2022; Widrich et al., 2020a). For example, for simulated repertoires, motif implants in $\geq$10% of sequences were required to affect the amino acid frequency and architecture features, suggesting that even in the case of high clonal expansion, the impact on the repertoire might not be sufficient to significantly change major repertoire

CellPress
OPEN ACCESS

features. This is reinforced by results showing that the disease-driving response in multiple autoimmune diseases is only to a small part antigen specific (Christophersen et al., 2019; Dahal-Koirala et al., 2022). More generally, our paper advances the state of the art of the immune repertoire field by changing the null hypothesis. Specifically, currently, the predominant thinking is that any immune state changes measurably the immune repertoire in a systematic fashion. Our paper challenges this view by finding that, *a priori*, we should not expect to see differences (Figures 4 and S10), and any substantial change must be proven. This change of perspective is highly valuable to the field as it pushes it toward more sensitive and robust approaches to immune repertoire and machine learning analysis (Arnaout et al., 2021; Kanduri et al., 2021; Slabodkin et al., 2021). Specifically, the usefulness of global features for diagnostics is severely limited, and to detect single-sequence-level differences (Emerson et al., 2017; Kanduri et al., 2021; Widrich et al., 2020a), single-sequence-level statistical and machine learning approaches are needed (Greiff et al., 2020; Schattgen et al., 2021).

In the future, ultra-deep (Briney et al., 2019; Soto et al., 2019, 2020) and population-wide, large-scale immune repertoire projects such as Human Vaccines Project (Crowe and Koff, 2015) may benefit from using immuneREF for identifying immune event-driven aberrations from a baseline repertoire similarity. Furthermore, large-scale database initiatives such as the iReceptor gateway (Corrie et al., 2018) may benefit from immuneREF functionality for on-the-fly computation of inter-dataset similarity.

Limitations of the study

Although we consider the usefulness of the six chosen features to be established (Figures 2 and S3G–S3J), we concede that the asymptotic nature of the sufficiency calculation leaves the door open to the introduction of additional features. The proposed set of immuneREF features denotes in this sense a minimally sufficient set for the analysis of immune repertoire datasets. It ensures sufficient coverage of the major variation-introducing aspects. It is for that reason we devised immuneREF as inherently modular, allowing single- and multi-feature analysis as well as encouraging the addition of new features relevant for particular problems such as transcriptome analysis (Schneider-Hohendorf et al., 2018; Figure S6), HLA typing for TCR studies (DeWitt et al., 2018; Emerson et al., 2017; Francis et al., 2021), single-cell omics information (Han et al., 2021; Setliff et al., 2019; Sturm et al., 2020; Yermanos et al., 2021), gene-specific substitution profiles for somatic hypermutation analysis (Sheng et al., 2017), lineage-specific information (Hoehn et al., 2016, 2021), and antigen-specific and antigen-associated motifs identified by sequence clustering and machine learning (Akbar et al., 2019; Dash et al., 2017; Friedensohn et al., 2020; Glanville et al., 2017; Greiff et al., 2017b; Horst et al., 2021; Mason et al., 2019; Mayer-Blackwell et al., 2021; Meysman et al., 2018; Quiniou et al., 2020; Sidhom et al., 2019; Wong et al., 2020; Yohannes et al., 2021). In particular, a future extension of immuneREF may be a feature that reliably identifies antigen-specific sequences, thus increasing the amount of immune information recovered. More generally, adult repertoires are very complex and contain hidden information of many antigens at different time points that might have been shared by different individuals. For instance, repertoire fingerprints of influenza infection might be present on most studied individuals and could explain the difficulty to distinguish healthy and diseased individuals. New features including (single-cell-based) antigen specificity patterns may help separate shared infection marks on the immune repertoire.

STAR★METHODS

Detailed methods are provided in the online version of this paper and include the following:

- KEY RESOURCES TABLE
- RESOURCE AVAILABILITY
  - ○ Lead contact
  - ○ Materials availability
  - ○ Data and code availability
- METHOD DETAILS
  - ○ immuneREF features
  - ○ immuneREF feature: Evenness profiles (state of clonal expansion)
  - ○ immuneREF feature: Positional amino acid frequencies
  - ○ immuneREF feature: Sequence similarity network architecture
  - ○ immuneREF feature: Repertoire overlap (convergence)
  - ○ immuneREF feature: Germline gene diversity
  - ○ immuneREF feature: Gapped k-mer occurrence
  - ○ immuneREF feature: Transcriptome integration
  - ○ Calculating repertoire similarities per feature
  - ○ Repertoire similarity – Condensing features into a composite network
  - ○ Mutual information
  - ○ Quantification of mutual information across ensembles of repertoire features
  - ○ Local repertoire similarity
  - ○ Simulation of adaptive immune receptor repertoires representing ground truth data
  - ○ Immune repertoire sequencing datasets
- QUANTIFICATION AND STATISTICAL ANALYSIS

SUPPLEMENTAL INFORMATION

Supplemental information can be found online at https://doi.org/10.1016/j.crmeth.2022.100269.

ACKNOWLEDGMENTS

Support was provided from The Helmsley Charitable Trust (#2019PG-T1D011 to V.G.), UiO World-Leading Research Community (to V.G.), UiO:LifeSciences Convergence Environment Immunolingo (to V.G. and G.K.S.), EU Horizon 2020 iReceptorplus (#825821) (to V.G.), a Research Council of Norway FRIPRO project (#300740 to V.G.), a Research Council of Norway IKTPLUSS project (#311341 to V.G. and G.K.S.), a Norwegian Cancer Society grant (#215817 to V.G.), Stiftelsen Kristian Gerhard Jebsen (K.G.Jebsen Coeliac Disease Research Centre) (to G.K.S.), the Swiss National Science Foundation (project 31003A to S.T.R), the Norwegian Research Council, Helse Sør-Øst, and the University of Oslo through the Centre for Molecular Medicine Norway (#187615 to M.L.K.).

AUTHOR CONTRIBUTIONS

V.G., C.R.W., and S.T.R. contributed to the conception of the work. V.G., S.T.R., and X.L. contributed to supervision of the study. C.R.W. and V.G. contributed to the study design and method development. C.R.W., T.R., L.W., W.Z., J.W., and M.L.K. contributed to data analysis. C.R.W., T.R., and V.G. contributed to the first draft of the manuscript. All authors reviewed the manuscript and approved the submitted work.

DECLARATION OF INTERESTS

V.G. declares advisory board positions in aiNET GmbH, Enpicom B.V., Specifica Inc., Adaptyv Biosystems, and EVQLV. V.G. is a consultant for Roche/Genentech.

Received: September 16, 2020
Revised: April 1, 2022
Accepted: July 19, 2022
Published: August 22, 2022

REFERENCES

Akbar, R., Robert, P.A., Pavlović, M., Jeliazkov, J.R., Snapkov, I., Slabodkin, A., Weber, C.R., Scheffer, L., Miho, E., Haff, I.H., et al. (2019). A compact vocabulary of paratope-epitope interactions enables predictability of antibody-antigen binding. Preprint at bioRxiv. https://doi.org/10.1101/759498.

Alon, U., Mokryn, O., and Hershberg, U. (2021). Using domain based latent personal analysis of B cell clone diversity patterns to identify novel Relationships between the B cell clone populations in different tissues. Front. Immunol. *12*, 642673.

Amoriello, R., Greiff, V., Aldinucci, A., Bonechi, E., Carnasciali, A., Peruzzi, B., Repice, A.M., Mariottini, A., Saccardi, R., Mazzanti, B., et al. (2020). The TCR repertoire reconstitution in multiple sclerosis: comparing one-shot and continuous immunosuppressive therapies. Front. Immunol. *11*, 559.

Amoriello, R., Chernigovskaya, M., Greiff, V., Carnasciali, A., Massacesi, L., Barilaro, A., Repice, A.M., Biagioli, T., Aldinucci, A., Muraro, P.A., et al. (2021). TCR repertoire diversity in multiple Sclerosis: high-dimensional bioinformatics analysis of sequences from brain, cerebrospinal fluid and peripheral blood. EBioMedicine *68*, 103429.

Arnaout, R.A., Prak, E.T.L., Schwab, N., Rubelt, F., Adaptive Immune Receptor Repertoire Community; Arnaout, R.A., Arora, R., Bashford-Rogers, R., Breden, F., Bukhari, S.A.C., et al. (2021). The future of blood testing is the immunome. Front. Immunol. *12*, 626793.

Arora, R., Burke, H.M., and Arnaout, R. (2018). Immunological diversity with similarity. Preprint at bioRxiv. https://doi.org/10.1101/483131.

Avnir, Y., Watson, C.T., Glanville, J., Peterson, E.C., Tallarico, A.S., Bennett, A.S., Qin, K., Fu, Y., Huang, C.-Y., Beigel, J.H., et al. (2016). IGHV1-69 polymorphism modulates anti-influenza antibody repertoires, correlates with IGHV utilization shifts and varies by ethnicity. Sci. Rep. *6*, 23876.

Barennes, P., Quiniou, V., Shugay, M., Egorov, E.S., Davydov, A.N., Chudakov, D.M., Uddin, I., Ismail, M., Oakes, T., Chain, B., et al. (2021). Benchmarking of T cell receptor repertoire profiling methods reveals large systematic biases. Nat. Biotechnol. *39*, 236–245.

Bashford-Rogers, R.J.M., Palser, A.L., Huntly, B.J., Rance, R., Vassiliou, G.S., Follows, G.A., and Kellam, P. (2013). Network properties derived from deep sequencing of human B-cell receptor repertoires delineate B-cell populations. Genome Res. *23*, 1874–1884.

Bashford-Rogers, R.J.M., Bergamaschi, L., McKinney, E.F., Pombal, D.C., Mescia, F., Lee, J.C., Thomas, D.C., Flint, S.M., Kellam, P., Jayne, D.R.W., et al. (2019). Analysis of the B cell receptor repertoire in six immune-mediated diseases. Nature *574*, 122–126.

Ben-Hamo, R., and Efroni, S. (2011). The whole-organism heavy chain B cell repertoire from Zebrafish self-organizes into distinct network features. BMC Syst. Biol. *5*, 27.

Bolen, C.R., Rubelt, F., Vander Heiden, J.A., and Davis, M.M. (2017). The repertoire dissimilarity index as a method to compare lymphocyte receptor repertoires. BMC Bioinf. *18*, 155.

Bolotin, D.A., Poslavsky, S., Mitrophanov, I., Shugay, M., Mamedov, I.Z., Putintseva, E.V., and Chudakov, D.M. (2015). MiXCR: software for comprehensive adaptive immunity profiling. Nat. Methods *12*, 380–381.

Briney, B., Inderbitzin, A., Joyce, C., and Burton, D.R. (2019). Commonality despite exceptional diversity in the baseline human antibody repertoire. Nature *566*, 393–397.

Brown, S.D., Raeburn, L.A., and Holt, R.A. (2015). Profiling tissue-resident T cell repertoires by RNA sequencing. Genome Med. *7*, 125.

Brown, A.J., Snapkov, I., Akbar, R., Pavlović, M., Miho, E., Sandve, G.K., and Greiff, V. (2019). Augmenting adaptive immunity: progress and challenges in the quantitative engineering and analysis of adaptive immune receptor repertoires. Mol. Syst. Des. Eng. *4*, 701–736.

Chiffelle, J., Genolet, R., Perez, M.A., Coukos, G., Zoete, V., and Harari, A. (2020). T-cell repertoire analysis and metrics of diversity and clonality. Curr. Opin. Biotechnol. *65*, 284–295.

Christophersen, A., Lund, E.G., Snir, O., Solà, E., Kanduri, C., Dahal-Koirala, S., Zühlke, S., Molberg, Ø., Utz, P.J., Rohani-Pichavant, M., et al. (2019). Distinct phenotype of CD4+ T cells driving celiac disease identified in multiple autoimmune conditions. Nat. Med. *25*, 734–737.

Clarke, E., and Sherrill-Mix, S. (2017). Ggbeeswarm: Categorical Scatter (Violin Point) Plot (R Foundation for Statistical Computing).

Cobey, S., Wilson, P., and Matsen, F.A. (2015). The evolution within us. Philos. Trans. R. Soc. Lond. B Biol. Sci. *370*, 20140235.

Corrie, B.D., Marthandan, N., Zimonja, B., Jaglale, J., Zhou, Y., Barr, E., Knoetze, N., Breden, F.M.W., Christley, S., Scott, J.K., et al. (2018). iReceptor: a platform for querying and analyzing antibody/B-cell and T-cell receptor repertoire data across federated repositories. Immunol. Rev. *284*, 24–41.

Cowell, L., Fonner, J., Jordan, C., Levin, M., Mock, S., Monson, N., Rounds, W., Salinas, E., Scarborough, W., and Scheuermann, R. (2015). VDJServer: a web-accessible analysis portal for immune repertoire sequence data (HUM1P.317). J. Immunol. *194*, 52.42.

Crowe, J.E., Jr., and Koff, W.C. (2015). Deciphering the human immunome. Expert Rev. Vaccines *14*, 1421–1425.

Csardi, G., and Nepusz, T. (2006). The igraph software package for complex network research. Int. J. Complex Syst., 1695.

Csepregi, L., Hoehn, K.B., Neumeier, D., Taft, J.M., Friedensohn, S., Weber, C.R., Kummer, A., Sesterhenn, F., Correia, B.E., and Reddy, S.T. (2021). The physiological landscape and specificity of antibody repertoires. Preprint at bioRxiv. https://doi.org/10.1101/2021.09.15.460420.

Dahal-Koirala, S., Balaban, G., Neumann, R.S., Scheffer, L., Lundin, K.E.A., Greiff, V., Sollid, L.M., Qiao, S.-W., and Sandve, G.K. (2022). TCRpower: quantifying the detection power of T-cell receptor sequencing with a novel computational pipeline calibrated by spike-in sequences. Brief. Bioinform., bbab566.

Dash, P., Fiore-Gartland, A.J., Hertz, T., Wang, G.C., Sharma, S., Souquette, A., Crawford, J.C., Clemens, E.B., Nguyen, T.H.O., Kedzierska, K., et al. (2017). Quantifiable predictive features define epitope-specific T cell receptor repertoires. Nature *547*, 89–93.

DeWitt, W.S., III, Smith, A., Schoch, G., Hansen, J.A., Matsen, F.A., Bradley, P., and Bradley, P. (2018). Human T cell receptor occurrence patterns encode immune history, genetic background, and receptor specificity. Elife *7*, e38358.

Emerson, R.O., DeWitt, W.S., Vignali, M., Gravley, J., Hu, J.K., Osborne, E.J., Desmarais, C., Klinger, M., Carlson, C.S., Hansen, J.A., et al. (2017). Immunosequencing identifies signatures of cytomegalovirus exposure history and HLA-mediated effects on the T cell repertoire. Nat. Genet. *49*, 659–665.

Folashade, D., Ooi, H., Calaway, R., and Microsoft; and Weston, S. (2019a). Foreach: Provides Foreach Looping Construct (R Foundation for Statistical Computing).

Folashade, D., and Revolution Analytics; and Weston, S. (2019b). doMC: Foreach Parallel Adaptor for "Parallel".

Francis, J.M., Leistritz-Edwards, D., Dunn, A., Tarr, C., Lehman, J., Dempsey, C., Hamel, A., Rayon, V., Liu, G., Wang, Y., et al. (2021). Allelic variation in class I HLA determines CD8+ T cell repertoire shape and cross-reactive memory responses to SARS-CoV-2. Sci. Immunol. *67*, eabk3070.

Friedensohn, S., Neumeier, D., Khan, T.A., Csepregi, L., Parola, C., Vries, A.R.G. de, Erlach, L., Mason, D.M., and Reddy, S.T. (2020). Convergent selection in antibody repertoires is revealed by deep learning. Preprint at bioRxiv. https://doi.org/10.1101/2020.02.25.965673.

Ghraichy, M., von Niederhäusern, V., Kovaltsuk, A., Galson, J.D., Deane, C.M., and Trück, J. (2021). Different B cell subpopulations show distinct patterns in their IgH repertoire metrics. Elife *10*, e73111.

Giudicelli, V., Chaume, D., and Lefranc, M.-P. (2004). IMGT/V-QUEST, an integrated software program for immunoglobulin and T cell receptor V-J and V-D-J rearrangement analysis. Nucleic Acids Res. *32*, W435–W440.

Glanville, J., Huang, H., Nau, A., Hatton, O., Wagar, L.E., Rubelt, F., Ji, X., Han, A., Krams, S.M., Pettus, C., et al. (2017). Identifying specificity groups in the T cell receptor repertoire. Nature *547*, 94–98.

Greiff, V., Miho, E., Menzel, U., and Reddy, S.T. (2015a). Bioinformatic and statistical analysis of adaptive immune repertoires. Trends Immunol. *36*, 738–749.

Gomez-Cabrero, D., Tarazona, S., Ferreirós-Vidal, I., Ramirez, R.N., Company, C., Schmidt, A., Reijmers, T., Paul, V.V.S., Marabita, F., Rodríguez-Ubreva, J., et al. (2019). STATegra, a comprehensive multi-omics dataset of B-cell differentiation in mouse. Sci. Data *6*, 256.

Greiff, V., Bhat, P., Cook, S.C., Menzel, U., Kang, W., and Reddy, S.T. (2015b). A bioinformatic framework for immune repertoire diversity profiling enables detection of immunological status. Genome Med. *7*, 49.

Greiff, V., Menzel, U., Miho, E., Weber, C., Riedel, R., Cook, S., Valai, A., Lopes, T., Radbruch, A., Winkler, T.H., and Reddy, S.T. (2017a). Systems analysis reveals high genetic and antigen-driven Predetermination of antibody repertoires throughout B cell development. Cell Rep. *19*, 1467–1478.

Greiff, V., Weber, C.R., Palme, J., Bodenhofer, U., Miho, E., Menzel, U., and Reddy, S.T. (2017b). Learning the high-dimensional immunogenomic features that Predict public and private antibody repertoires. J. Immunol. *199*, 2985–2997.

Greiff, V., Yaari, G., and Cowell, L. (2020). Mining adaptive immune receptor repertoires for biological and clinical information using machine learning. Curr. Opin. Syst. Biol. *24*, 109–119.

Gu, Z. (2015). ComplexHeatmap: making complex heatmaps. Bioinformatics. https://doi.org/10.18129/B9.bioc.ComplexHeatmap.

Gupta, N.T., Heiden, J.V., Uduman, M., Gadala-Maria, D., Yaari, G., and Kleinstein, S.H. (2015). Change-O: a toolkit for analyzing large-scale B cell immunoglobulin repertoire sequencing data. Bioinformatics *31*, 3356–3358.

Hackstadt, A.J., and Hess, A.M. (2009). Filtering for increased power for microarray data analysis. BMC Bioinf. *10*, 11.

Han, J., Kuhn, R., Papadopoulou, C., Agrafiotis, A., Kreiner, V., Shlesinger, D., Dizerens, R., Hong, K.-L., Weber, C., Greiff, V., et al. (2021). Echidna: integrated simulations of single-cell immune receptor repertoires and transcriptomes. Preprint at bioRxiv. https://doi.org/10.1101/2021.07.17.452792.

Vander Heiden, J.A., Yaari, G., Uduman, M., Stern, J.N., O'Connor, K.C., Hafler, D.A., Vigneault, F., and Kleinstein, S.H. (2014). pRESTO: a toolkit for processing high-throughput sequencing raw reads of lymphocyte receptor repertoires. Bioinformatics *30*, 1930–1932.

Hoehn, K.B., Fowler, A., Lunter, G., and Pybus, O.G. (2016). The diversity and molecular evolution of B-cell receptors during infection. Mol. Biol. Evol. *33*, 1147–1157.

Hoehn, K.B., Turner, J.S., Miller, F.I., Jiang, R., Pybus, O.G., Ellebedy, A.H., and Kleinstein, S.H. (2021). Human B cell lineages associated with germinal centers following influenza vaccination are measurably evolving. Elife *10*, e70873.

Horst, A., Smakaj, E., Natali, E.N., Tosoni, D., Babrak, L.M., Meier, P., and Miho, E. (2021). Machine learning detects anti-DENV signatures in antibody repertoire sequences. Front. Artif. Intell. *4*, 715462.

Johnson, S.A., Seale, S.L., Gittelman, R.M., Rytlewski, J.A., Robins, H.S., and Fields, P.A. (2021). Impact of HLA type, age and chronic viral infection on peripheral T-cell receptor sharing between unrelated individuals. PLoS One *16*, e0249484.

Kanduri, C., Pavlović, M., Scheffer, L., Motwani, K., Chernigovskaya, M., Greiff, V., and Sandve, G.K. (2021). Profiling the baseline performance and limits of machine learning models for adaptive immune receptor repertoire classification. Preprint at bioRxiv. https://doi.org/10.1101/2021.05.23.445346.

Kaplinsky, J., and Arnaout, R. (2016). Robust estimates of overall immune-repertoire diversity from high-throughput measurements on samples. Nat. Commun. *7*, 11881.

Kovaltsuk, A., Leem, J., Kelm, S., Snowden, J., Deane, C.M., and Krawczyk, K. (2018). Observed antibody space: a resource for data mining next-Generation sequencing of antibody repertoires. J. Immunol. *201*, 2502–2509.

Li, H., Limenitakis, J.P., Greiff, V., Yilmaz, B., Schären, O., Urbaniak, C., Zünd, M., Lawson, M.A.E., Young, I.D., Rupp, S., et al. (2020). Mucosal or systemic microbiota exposures shape the B cell repertoire. Nature *584*, 274–278.

Liu, X., Zhang, W., Zhao, M., Fu, L., Liu, L., Wu, J., Luo, S., Wang, L., Wang, Z., Lin, L., et al. (2019). T cell receptor β repertoires as novel diagnostic markers for systemic lupus erythematosus and rheumatoid arthritis. Ann. Rheum. Dis. *78*, 1070–1078.

Marcou, Q., Mora, T., and Walczak, A.M. (2017). IGoR: a Tool for high-throughput immune repertoire analysis. Preprint at bioRxiv, 141143. https://doi.org/10.1101/141143.

Mason, D.M., Friedensohn, S., Weber, C.R., Jordi, C., Wagner, B., Meng, S., Gainza, P., Correia, B.E., and Reddy, S.T. (2019). Deep learning enables therapeutic antibody optimization in mammalian cells by deciphering high-dimensional protein sequence space. Preprint at bioRxiv. https://doi.org/10.1101/617860.

Mason, D.M., Weber, C.R., Parola, C., Meng, S.M., Greiff, V., Kelton, W.J., and Reddy, S.T. (2018). High-throughput antibody engineering in mammalian cells by CRISPR/Cas9-mediated homology-directed mutagenesis. Nucleic Acids Res. *46*, 7436–7449.

Mayer-Blackwell, K., Schattgen, S., Cohen-Lavi, L., Crawford, J.C., Souquette, A., Gaevert, J.A., Hertz, T., Thomas, P.G., Bradley, P., and Fiore-Gartland, A. (2021). TCR meta-clonotypes for biomarker discovery with tcrdist3: identification of public, HLA-restricted SARS-CoV-2 associated TCR features. Preprint at bioRxiv. https://doi.org/10.1101/2020.12.24.424260.

Meysman, P., De Neuter, N., Gielis, S., Bui Thi, D., Ogunjimi, B., and Laukens, K. (2018). On the viability of unsupervised T-cell receptor sequence clustering for epitope preference. Bioinformatics *35*, 1461–1468.

Miho, E., Yermanos, A., Weber, C.R., Berger, C.T., Reddy, S.T., and Greiff, V. (2018). Computational strategies for dissecting the high-dimensional complexity of adaptive immune repertoires. Front. Immunol. *9*, 224.

Miho, E., Roškar, R., Greiff, V., and Reddy, S.T. (2019). Large-scale network analysis reveals the sequence space architecture of antibody repertoires. Nat. Commun. *10*, 1321.

Minervina, A.A., Komech, E.A., Titov, A., Bensouda Koraichi, M., Rosati, E., Mamedov, I.Z., Franke, A., Efimov, G.A., Chudakov, D.M., Mora, T., et al. (2021). Longitudinal high-throughput TCR repertoire profiling reveals the dynamics of T cell memory formation after mild COVID-19 infection. Elife *10*, e63502.

Moon, K.W. (2018). ggiraphExtra: Make Interactive 'ggplot2'. Extension to 'ggplot2' and 'ggiraph' (R Foundation for Statistical Computing).

Nazarov, V., and immunarch.bot; and Rumynskiy, E. (2020). immunomind/immunarch: 0.6.5: Basic Single-Cell Support (Zenodo).

Neuwirth, E. (2014). RColorBrewer: ColorBrewer Palettes (R Foundation for Statistical Computing).

Olson, B.J., Moghimi, P., Schramm, C., Obraztsova, A., Ralph, D., Heiden, J.A.V., Shugay, M., Shepherd, A., Lees, W., and Matsen, F.A. (2019). sumrep: a summary statistic framework for immune receptor repertoire comparison and model validation. Preprint at bioRxiv. https://doi.org/10.1101/727784.

Ota, M., Nakano, M., Nagafuchi, Y., Kobayashi, S., Hatano, H., Yoshida, R., Akutsu, Y., Itamiya, T., Matsuo, A., Tsuchida, Y., et al. (2022). Multimodal repertoire analysis unveils B cell biology in health and immune-mediated. Preprint at medRxiv. https://doi.org/10.1101/2022.01.04.22268769.

Palme, J., Hochreiter, S., and Bodenhofer, U. (2015). KeBABS: an R package for kernel-based analysis of biological sequences: Fig. 1. Bioinformatics *31*, 2574–2576.

Pancer, Z., and Cooper, M.D. (2006). The evolution of adaptive immunity. Annu. Rev. Immunol. *24*, 497–518.

Pavlović, M., Scheffer, L., Motwani, K., Kanduri, C., Kompova, R., Vazov, N., Waagan, K., Bernal, F.L.M., Costa, A.A., Corrie, B., et al. (2021). The immuneML ecosystem for machine learning analysis of adaptive immune receptor repertoires. Nat. Mach. Intell. *3*, 936–944.

Pertseva, M., Gao, B., Neumeier, D., Yermanos, A., and Reddy, S.T. (2021). Applications of machine and deep learning in adaptive immunity. Annu. Rev. Chem. Biomol. Eng. *12*, 39–62.

Pogorelyy, M.V., and Shugay, M. (2019). A framework for Annotation of antigen Specificities in high-throughput T-cell repertoire sequencing studies. Front. Immunol. *10*, 2159.

Pogorelyy, M.V., Minervina, A.A., Shugay, M., Chudakov, D.M., Lebedev, Y.B., Mora, T., and Walczak, A.M. (2019). Detecting T cell receptors involved in immune responses from single repertoire snapshots. PLoS Biol. *17*, e3000314.

Quiniou, V., Barennes, P., Martina, F., Mhanna, V., Vantomme, H., Pham, H.P., Shugay, M., Six, A., Mariotti-Ferrandiz, E., and Klatzmann, D. (2020). Human thymopoiesis selects unconventional CD8+ α/β T cells that respond to multiple viruses. Preprint at bioRxiv. https://doi.org/10.1101/2020.07.27.223354.

R Core Team (2013). R: A Language and Environment for Statistical Computing (R Foundation for Statistical Computing).

Raybould, M.I.J., Marks, C., Krawczyk, K., Taddese, B., Nowak, J., Lewis, A.P., Bujotzek, A., Shi, J., and Deane, C.M. (2019). Five computational developability guidelines for therapeutic antibody profiling. Proc. Natl. Acad. Sci. USA. *116*, 4025–4030.

Raybould, M.I.J., Rees, A.R., and Deane, C.M. (2021). Current strategies for detecting functional convergence across B-cell receptor repertoires. mAbs *13*, 1996732.

Riedel, R., Addo, R., Ferreira-Gomes, M., Heinz, G.A., Heinrich, F., Kummer, J., Greiff, V., Schulz, D., Klaeden, C., Cornelis, R., et al. (2020). Discrete populations of isotype-switched memory B lymphocytes are maintained in murine spleen and bone marrow. Nat. Commun. *11*, 2570.

Rognes, T., Scheffer, L., Greiff, V., and Sandve, G.K. (2022). CompAIRR: ultrafast comparison of adaptive immune receptor repertoires by exact and approximate sequence matching. Bioinformatics. https://doi.org/10.1093/bioinformatics/btac505.

Rosati, E., Pogorelyy, M.V., Minervina, A.A., Franke, A., Scheffold, A., Bacher, P., and Thomas, P. (2021). Characterization of SARS-CoV-2 public CD4+ $\alpha\beta$ T cell clonotypes through reverse epitope discovery. Preprint at bioRxiv. https://doi.org/10.1101/2021.11.19.469229.

Rubelt, F., Busse, C.E., Bukhari, S.A.C., Bürckert, J.-P., Mariotti-Ferrandiz, E., Cowell, L.G., Watson, C.T., Marthandan, N., Faison, W.J., Hershberg, U., et al. (2017). Adaptive immune receptor repertoire community recommendations for sharing immune-repertoire sequencing data. Nat. Immunol. *18*, 1274–1278.

Rubio, T., Chernigovskaya, M., Marquez, S., Marti, C., Izquierdo-Altarejos, P., Urios, A., Montoliu, C., Felipo, V., Conesa, A., Greiff, V., and Tarazona, S. (2022). A Nextflow pipeline for T-cell receptor repertoire reconstruction and analysis from RNA sequencing data. ImmunoInformatics *6*, 100012.

Safonova, Y., Lapidus, A., and Lill, J. (2015). IgSimulator: a versatile immunosequencing simulator. Bioinformatics *31*, 3213–3215.

Schattgen, S.A., Guion, K., Crawford, J.C., Souquette, A., Barrio, A.M., Stubbington, M.J.T., Thomas, P.G., and Bradley, P. (2021). Integrating T cell receptor sequences and transcriptional profiles by clonotype neighbor graph analysis (CoNGA). Nat. Biotechnol. *40*, 54–63.

Schloerke, B., Crowley, J., Cook, D., Briatt, F., Marbach, M., Thoen, E., Amos, E., and Joseph, L. (2018). GGally: Extension to ‘Ggplot2’ (R Foundation for Statistical Computing).

Schneider-Hohendorf, T., Görlich, D., Savola, P., Kelkka, T., Mustjoki, S., Gross, C.C., Owens, G.C., Klotz, L., Dornmair, K., Wiendl, H., and Schwab, N. (2018). Sex bias in MHC I-associated shaping of the adaptive immune system. Proc. Natl. Acad. Sci. USA. *115*, 2168–2173.

Setliff, I., Shiakolas, A.R., Pilewski, K.A., Murji, A.A., Mapengo, R.E., Janowska, K., Richardson, S., Oosthuysen, C., Raju, N., Ronsard, L., et al. (2019). High-throughput mapping of B cell receptor sequences to antigen specificity. Cell *179*, 1636–1646.e15.

Shemesh, O., Polak, P., Lundin, K.E.A., Sollid, L.M., and Yaari, G. (2021). Machine learning analysis of naïve B-cell receptor repertoires stratifies celiac disease patients and controls. Front. Immunol. *12*, 627813.

Sheng, Z., Schramm, C.A., Kong, R., NISC Comparative Sequencing Program; Mullikin, J.C., Mascola, J.R., Kwong, P.D., Shapiro, L., Benjamin, B., Bouffard, G., et al. (2017). Gene-specific substitution profiles describe the types and frequencies of amino acid changes during antibody somatic hypermutation. Front. Immunol. *8*, 537.

Shugay, M., Bagaev, D.V., Turchaninova, M.A., Bolotin, D.A., Britanova, O.V., Putintseva, E.V., Pogorelyy, M.V., Nazarov, V.I., Zvyagin, I.V., Kirgizova, V.I., et al. (2015). VDJtools: Unifying post-analysis of T cell receptor repertoires. PLoS Comput. Biol. *11*, e1004503.

Sidhom, J.-W., Larman, H.B., Ross-MacDonald, P., Wind-Rotolo, M., Pardoll, D.M., and Baras, A.S. (2019). DeepTCR: a deep learning framework for understanding T-cell receptor sequence signatures within complex T-cell repertoires. Preprint at bioRxiv. https://doi.org/10.1101/464107.

Slabodkin, A., Chernigovskaya, M., Mikocziova, I., Akbar, R., Scheffer, L., Pavlović, M., Bashour, H., Snapkov, I., Mehta, B.B., Weber, C.R., et al. (2021). Individualized VDJ recombination predisposes the available Ig sequence space. Preprint at bioRxiv. https://doi.org/10.1101/2021.04.19.440409.

Song, L., Cohen, D., Ouyang, Z., Cao, Y., Hu, X., and Liu, X.S. (2021). TRUST4: immune repertoire reconstruction from bulk and single-cell RNA-seq data. Nat. Methods *18*, 627–630.

Soto, C., Bombardi, R.G., Branchizio, A., Kose, N., Matta, P., Sevy, A.M., Sinkovits, R.S., Gilchuk, P., Finn, J.A., and Crowe, J.E. (2019). High frequency of shared clonotypes in human B cell receptor repertoires. Nature *566*, 398–402.

Soto, C., Bombardi, R.G., Kozhevnikov, M., Sinkovits, R.S., Chen, E.C., Branchizio, A., Kose, N., Day, S.B., Pilkinton, M., Gujral, M., et al. (2020). High frequency of shared clonotypes in human T cell receptor repertoires. Cell Rep. *32*, 107882.

Stern, J.N.H., Yaari, G., Vander Heiden, J.A., Church, G., Donahue, W.F., Hintzen, R.Q., Huttner, A.J., Laman, J.D., Nagra, R.M., Nylander, A., et al. (2014). B cells populating the multiple sclerosis brain mature in the draining cervical lymph nodes. Sci. Transl. Med. *6*, 248ra107.

Strauli, N.B., and Hernandez, R.D. (2016). Statistical inference of a convergent antibody repertoire response to influenza vaccine. Genome Med. *8*, 60.

Sturm, G., Szabo, T., Fotakis, G., Haider, M., Rieder, D., Trajanoski, Z., and Finotello, F. (2020). Scirpy: a scanpy extension for analyzing single-cell T-cell receptor-sequencing data. Bioinformatics *36*, 4817–4818.

Thomas, N., Best, K., Cinelli, M., Reich-Zeliger, S., Gal, H., Shifrut, E., Madi, A., Friedman, N., Shawe-Taylor, J., and Chain, B. (2014). Tracking global changes induced in the CD4 T-cell receptor repertoire by immunization with a complex antigen using short stretches of CDR3 protein sequence. Bioinformatics *30*, 3181–3188.

Vujović, M., Marcatili, P., Chain, B., Kaplinsky, J., and Andresen, T.L. (2021). T-cell receptor diversity estimates for repertoires (TCRDivER) uses sequence similarity to find signatures of immune response. Preprint at bioRxiv. https://doi.org/10.1101/2021.01.11.417444.

Wang, B., Mezlini, A.M., Demir, F., Fiume, M., Tu, Z., Brudno, M., Haibe-Kains, B., and Goldenberg, A. (2014). Similarity network fusion for aggregating data types on a genomic scale. Nat. Methods *11*, 333–337.

Weber, C.R., Akbar, R., Yermanos, A., Pavlović, M., Snapkov, I., Sandve, G.K., Reddy, S.T., and Greiff, V. (2019). immuneSIM: tunable multi-feature simulation of B- and T-cell receptor repertoires for immunoinformatics benchmarking. Preprint at bioRxiv. https://doi.org/10.1101/759795.

Weber, C.R., Akbar, R., Yermanos, A., Pavlović, M., Snapkov, I., Sandve, G.K., Reddy, S.T., and Greiff, V. (2020). immuneSIM: tunable multi-feature simulation of B- and T-cell receptor repertoires for immunoinformatics benchmarking. Bioinformatics *36*, 3594–3596.

Wickham, H. (2009). ggplot2: Elegant Graphics for Data Analysis (Springer-Verlag).

Widrich, M., Schäfl, B., Pavlović, M., Sandve, G.K., Hochreiter, S., Greiff, V., and Klambauer, G. (2020a). DeepRC: immune repertoire classification with attention-based deep massive multiple instance learning. Preprint at bioRxiv. https://doi.org/10.1101/2020.04.12.038158.

Widrich, M., Schäfl, B., Ramsauer, H., Pavlović, M., Gruber, L., Holzleitner, M., Brandstetter, J., Sandve, G.K., Greiff, V., Hochreiter, S., et al. (2020b). Modern hopfield networks and attention for immune repertoire classification. Preprint at arXiv. https://doi.org/10.48550/arXiv.2007.13505.

Wong, W.K., Robinson, S.A., Bujotzek, A., Georges, G., Lewis, A.P., Shi, J., Snowden, J., Taddese, B., and Deane, C. (2020). Ab-Ligity: identifying sequence-dissimilar antibodies that bind to the same epitope. Preprint at bioRxiv. https://doi.org/10.1101/2020.03.24.004051.

Yaari, G., and Kleinstein, S.H. (2015). Practical guidelines for B-cell receptor repertoire sequencing analysis. Genome Med. *7*, 121.

Yermanos, A., Agrafiotis, A., Kuhn, R., Robbiani, D., Yates, J., Papadopoulou, C., Han, J., Sandu, I., Weber, C., Bieberich, F., et al. (2021). Platypus: an open-access software for integrating lymphocyte single-cell immune repertoires with transcriptomes. NAR Genom. Bioinform. *3*, lqab023.

Yohannes, D.A., Kaukinen, K., Kurppa, K., Saavalainen, P., and Greco, D. (2021). Clustering based approach for population level identification of condition-associated T-cell receptor β-chain CDR3 sequences. BMC Bioinf. *22*, 159.

Zhang, W., Wang, L., Liu, K., Wei, X., Yang, K., Du, W., Wang, S., Guo, N., Ma, C., Luo, L., et al. (2019). PIRD: pan immune repertoire database. Bioinformatics *36*, 897–903.

STAR★METHODS

KEY RESOURCES TABLE

REAGENT or RESOURCE	SOURCE	IDENTIFIER
**Deposited data**		
Mouse (C57/BL6J), B cell	Greiff et al., 2017a	E-MTAB-5349
Human (CMV dataset), T cell	Emerson et al., 2017	https://doi.org/10.21417/B7001Z
Human (Covid dataset), T cell	Minervina et al., 2021	PRJNA633317
Human (Autoimmune dataset), T cell	Zhang et al., 2019	https://db.cngb.org/pird/PIRD. Project IDs: P18081001, P18081101, P18080801.
**Software and algorithms**		
R statistical programming environment	https://www.r-project.org/	v3.6.1
ggplot2	https://ggplot2.tidyverse.org/	R package version 3.2.1
Ggbeeswarm	https://cran.r-project.org/web/packages/ggbeeswarm/	R package version 0.6.0
RColorBrewer	https://cran.r-project.org/web/packages/RColorBrewer/index.html	R package version 1.1-2
Igraph	https://cran.r-project.org/web/packages/igraph/index.html	R package version 1.2.4.2
ggiraphExtra	https://cran.r-project.org/web/packages/ggiraphExtra/vignettes/introduction.html	R package version 0.2.9
GGally	https://cran.r-project.org/web/packages/GGally/index.html	R package version 1.4.0
Foreach	https://cran.r-project.org/web/packages/foreach/index.html	R package version 1.4.7
doMC	https://cran.r-project.org/web/packages/doMC/index.html	R package version 1.3.6
ComplexHeatmap	https://www.bioconductor.org/packages/release/bioc/html/ComplexHeatmap.html	R package version 2.2.0
immuneREF	this paper	https://doi.org/10.5281/zenodo.5522406

RESOURCE AVAILABILITY

Lead contact

Further information and requests for resources and reagents should be directed to and will be fulfilled by the lead contact, Victor Greiff (victor.greiff@medisin.uio.no).

Materials availability

This study did not generate new unique reagents.

Data and code availability

- Data: This paper analyzes existing, publicly available data. These accession numbers for the datasets are listed in the Key Resources Table.
- Code: The immuneREF analysis workflow is made available via the immuneREF R package hosted on GitHub (https://github.com/GreiffLab/immuneREF). Documentation of the immuneREF package is provided on readthedocs (https://immuneref.readthedocs.io).
- Any additional information required to reanalyze the data reported in this work paper is available from the Lead contact upon request.

METHOD DETAILS

immuneREF features

For each dataset, we calculated six immune repertoire features and a per-feature similarity score.

immuneREF feature: Evenness profiles (state of clonal expansion)

Evenness profiles were calculated as described previously (Greiff et al., 2015b) on the CDR3 nucleotide level. Briefly, we calculated the Hill-diversity for alpha values 0–10 in steps of 0.1 with alpha = 1 being defined as the Shannon evenness. Each entry in the profile varies between ≈ 0 and 1, where higher values indicate an increasingly uniform clonal frequency distribution. We determined evenness profiles for each repertoire and evaluated cross-repertoire evenness similarity by Pearson correlation of the repertoires' evenness profiles as described previously (Amoriello et al., 2020; Greiff et al., 2015b, 2017a).

immuneREF feature: Positional amino acid frequencies

The positional amino acid frequencies were calculated separately for each CDR3 sequence length. To decrease bias by extraordinarily short or long CDR3 sequences, we limited this analysis to a range of the most common lengths (8–20 amino acids) (Greiff et al., 2017a; Raybould et al., 2019). Briefly, per position amino acid frequencies were calculated for each length. Subsequently, the resulting per length frequency vectors of each repertoire were Pearson-correlated by length and the mean correlation was calculated. Unlike in the case of k-mer occurrences, no positions are excluded, making AA frequency more sensitive to VDJ usage perturbations. Relative frequencies were used for all positional amino acid frequency calculations.

immuneREF feature: Sequence similarity network architecture

As previously described (Miho et al., 2019), we constructed a sequence similarity network for each immune repertoire: nodes represent amino acid CDR3 sequences connected by similarity edges if they had a Levenshtein Distance of 1 (LD = 1). The igraph R package was used to calculate networks (v.1.2.4.1, Csardi and Nepusz, 2006), that were analyzed with respect to four measures representing different aspects of network architecture: (i) cumulative degree distribution, (ii) mean hub score (Kleinberg hub centrality score), (iii) fraction of unconnected clusters and nodes and (iv) percent of sequences in the largest connected component. An LD = 1 network was constructed for each repertoire and the similarity between the repertoires' resulting network was evaluated with respect to their differences in the cumulative degree distribution, mean hub-score, outlier sequence occurrence, and largest network components; these metrics have been shown to be defining repertoire characteristics that are robust to subsampling (Miho et al., 2019). The similarity of the architecture between two repertoires A and B was calculated as the mean of four components: (i) the cumulative degree distribution (Pearson correlation between repertoires), (ii) mean hub scores $(1 - |MeanHubScore_A - |MeanHubScore_B)$, (iii) the fraction of unconnected components, and (iv) the fraction of sequences in the largest component $(1 - |PercLargestComponents_A - PercLargestComponents_B|)$. Unlike many of the other features, the network feature combines multiple single measures, which rendered it difficult to perform Pearson correlation analysis involving all four investigated network measures. Therefore, we adopted the network feature comparison approach described above.

immuneREF feature: Repertoire overlap (convergence)

The pairwise repertoire clonal overlap (clones defined based on 100% similarity of CDR3 amino acid sequence), was calculated across repertoires, as previously described (Greiff et al., 2017b):

$$\text{overlap}\ (X, Y) = \frac{|X \cap Y|}{\min(|X|, |Y|)} \times 100 \ \text{ where } \ X = \text{repertoire} \ X, Y = \text{Repertoire} \ Y$$

This clonal sequence overlap measure represents the similarity value between repertoires with respect to clonal convergence.

immuneREF feature: Germline gene diversity

The relative frequency of germline genes (defined by the ImmunoGenetics Database, IMGT) (Giudicelli et al., 2004) across clones in each repertoire was calculated for each repertoire depending on species and immune receptor class (Ig, TR). The germline gene usage allows insight into deviations from a baseline recombinational likelihood and thereby captures the potential impact of disease, vaccine, or other events on the immune state (Avnir et al., 2016; Greiff et al., 2017a). To determine germline gene usage similarities, we examined the V- and J-gene frequencies across clones for each individual. The Pearson correlation coefficient was determined for each of the frequency vectors (V-, D-, J-gene) with entries of all IMGT variants in a pairwise fashion between samples as described previously (Greiff et al., 2017a; Weber et al., 2019). Specifically, the correlations are calculated per germline gene, leading to separate V_cor, D_cor, J_cor values (and additionally VJ_cor for each V_J combination). The resulting correlation values are combined into a single value by calculating a weighted mean of these components. The weight vector used for the results in the manuscript is c(V = 1,D = 1,J = 1,VJ = 0).

immuneREF feature: Gapped k-mer occurrence

For a given k-mer size *k* and maximal gap length *m*, the nucleotide-based gapped-pair-k-mer occurrences were counted for all *gap sizes* $\leq m$ (Palme et al., 2015). The parameters *k* and *m* were chosen based on previous research (Greiff et al., 2017b), where defining parameters k = 3, m $\leq$ 3 was shown to lead to an encoding sufficient for sequence classification. The counts were normalized by the total number of gapped k-mers found across all gap sizes such that short-gap gapped-k-mers were weighted higher than larger gap sizes. While the amino acid frequency distribution contains positional information, the gapped k-mer occurrence represents short- and long-range sequential information encoded in the repertoire. We counted the occurrence of gapped k-mers (k = 3, m $\leq$ 3) across

all CDR3 sequences of a repertoire and correlated the resulting distributions between repertoire pairs using Pearson correlation as described previously (Weber et al., 2019).

immuneREF feature: Transcriptome integration

In order to keep the most informative genes from the genes obtained in a transcriptome experiment, immuneREF firstly applies a low variation filter (Hackstadt and Hess, 2009). Specifically, the standard deviation (SD) is calculated per gene across samples, and all genes above a certain threshold (default, SD > 1) are preserved for subsequent analysis.

To construct the gene expression feature similarity matrix, the Pearson correlation was calculated between samples. Additional approaches for the calculation of the gene expression feature similarity matrix implemented in the immuneREF package (mutual rank, PCA) are described in the package documentation.

Calculating repertoire similarities per feature

The calculation of the similarity values between a pair of repertoires was performed in a feature-specific manner as described in the methods section of each feature.

Repertoire similarity – Condensing features into a composite network

The single features are condensed into a multi-feature network by taking the mean of all single-feature similarity values resulting in a single repertoire similarity value. The resulting condensed network represents a weighted composite of the single-feature similarity networks. Additional approaches to obtain a composite network (max similarity, min similarity, SNF (Wang et al., 2014)) are implemented in the R-package as described in the package documentation.

Mutual information

Mutual information is a measure that quantifies to what extent one random variable explains another. Mutual information was defined as

$$I(X;Y) = H(X) - H(X|Y) = \sum_{x,y} P_{XY}(x,y) \log \frac{P_{XY}(x,y)}{P_x(x)P_y(y)}$$

Where, H(X) is the marginal entropy, H(X|Y) the conditional entropy P_{XY} the joint probability distribution of X and Y and P_X and P_Y the respective marginals. Mutual information was calculated using the R packages entropy (v.1.2.1, Hausser, 2014) and infotheo (v.1.2.0, Meyer, 2014). The values were normalized to the range [0,1] by dividing the mutual information by the sum of the entropies H(X)+H(Y). This normalized mutual information, also known as redundancy, is zero when both are independent and maximal when knowledge of one of the variables becomes redundant given the other.

Quantification of mutual information across ensembles of repertoire features

The mutual information between two features was calculated across all values in the similarity matrix, whereas the similarity matrix represents all pairwise similarity values between repertoires for a given feature. For the V(D)J diversity feature, values were set to zero by definition (i.e., the similarity between repertoires of different species/receptors) and were excluded from this calculation.

We ensembled immune information captured by the repertoire features (Figure 2) as the extent to which repertoire features collectively cover immune repertoire complexity. Specifically, we evaluated the change in mutual information between subsequently added features. Features were added one by one (*1*-feature network → *2*-feature network, *2*-feature network → *3*-feature network, and so forth, where *n*-feature means *n* features combined into a composite network), with the next feature to be chosen randomly (500 permutations of feature combinations per "n-features → *n+1*-feature" step).

Local repertoire similarity

To determine a single value measure for how connected a repertoire is within a subgraph (e.g. the repertoires of healthy human IgH repertoires and the similarity values between them), we defined the local similarity measure. It is calculated by dividing the node strength of each repertoire within a subgraph (sum of all edge weights connecting it to the other nodes in the subgraph) by the sum of all node strengths in the subgraph.

Local similarity gives the ratio of node strength that is connected to each repertoire in a subgraph and thus allows the identification of the most and least representative node of any category (the one most and least strongly connected within that category, respectively, see Figure 2C). The local similarity is dependent on the number of nodes within the subgraph and is therefore only used to compare repertoires within the subgraph. To enable comparison of local similarity values across different subgraphs, local similarity can be scaled by dividing by the number of nodes in the subgraph to correct for varying subgraph sizes in cases where the number of repertoires per subgraph differs.

Simulation of adaptive immune receptor repertoires representing ground truth data

We simulated 200 immune repertoire datasets where we controlled 40 parameter combinations over multiple replicates, thus allowing us to generate datasets where there is *ground truth*. Simulated repertoires were generated by the immuneSIM framework (R package) (v.0.8.7, Weber et al., 2019). Each simulated repertoire contained 12′000 sequences and varied with respect to species (mouse, human), receptor (BCR, TCR), germline gene distribution, clone count distribution, the occurrence of N1, N2 insertions and deletions in V, D, and J genes. Additionally, a subset of repertoires was modified post-simulation: in order to simulate motif occurrence, the motifs "YAY" ("tacgcctac") and "YVY" ("tacgtctac") were implanted with a probability of 2.5% each at a random position in the complementarity determining region (CDR3). To create repertoires with variation in sequence similarity network architecture, the top 5% sequences with the highest hub scores in a given repertoire were removed. In order to evaluate the sensitivity of the gapped k-mer occurrence feature, repertoires that differ in nucleotide composition, while retaining amino acid composition, were generated by introducing synonymous codons (“tat” → ”tac” for Tyrosine, “agt” → "agc" for Serine and ”gtt” → “gtg” for Valine) in 50% of VDJ sequences. Finally, the simulated and modified repertoires were subsampled to 10′000 sequences to ensure equal dataset size. The simulation parameters and their expected impact on each feature are summarized in Table S1.

Immune repertoire sequencing datasets

We conducted our analysis on 2′408 deep sequencing immune repertoires collected from four different studies: (i) a mouse immunization study of BCRs (flow cytometry-sorted B cells from different tissues: naive B cells from spleen (IgM), pre B cells (IgM) and IgG plasma cells from bone marrow, RNA-based high-throughput sequencing, preprocessed with MiXCR (Bolotin et al., 2015), for more details, please see (Greiff et al., 2017a)), (ii) a study of human TCRβ repertoires and signatures of cytomegalovirus, DNA-based high-throughput sequencing (CMV$^{+/-}$, unsorted PBMC) (Emerson et al., 2017), (iii) a study of TCR repertoires of patients recovered from mild cases of Covid-19 (Minervina et al., 2021) and (iv) the PanImmune repertoire database (PIRD, unsorted PBMC, preprocessed with iMonitor) (Zhang et al., 2019) (see Table S2). All sequences with stop codons were excluded and the naming of columns and V,D,J calls was standardized according to AIRR-community standards (Rubelt et al., 2017). When larger, each dataset was subsampled to 10′000 sequences (top clones by descending clonal frequency). Quality and read statistics may be found in the respective publications.

QUANTIFICATION AND STATISTICAL ANALYSIS

Statistical analysis was performed using R 3.6.1 (R Core Team, 2013). Graphics were generated using the R packages ggplot2 v3.2.1 (Wickham, 2009), ggbeeswarm v0.6.0 (Clarke and Sherrill-Mix, 2017), RColorBrewer v1.1-2 (Neuwirth, 2014), ComplexHeatmap v2.2.0 (heatmaps) (Gu, 2015), igraph v1.2.4.2 (network plots) (Csardi and Nepusz, 2006), ggiraphExtra v.0.2.9 (radar plots) (Moon, 2018), GGally v1.4.0 (parallel plots) (Schloerke et al., 2018). Parallel computing immuneREF analysis was performed using the R packages foreach v1.4.7 (Folashade et al., 2019a) and doMC v1.3.6 (Folashade et al., 2019b). Figure 1 was created using Biorender.com.

Cell Reports Methods, Volume *2*

Supplemental information

Reference-based comparison of adaptive immune receptor repertoires

Cédric R. Weber, Teresa Rubio, Longlong Wang, Wei Zhang, Philippe A. Robert, Rahmad Akbar, Igor Snapkov, Jinghua Wu, Marieke L. Kuijjer, Sonia Tarazona, Ana Conesa, Geir K. Sandve, Xiao Liu, Sai T. Reddy, and Victor Greiff

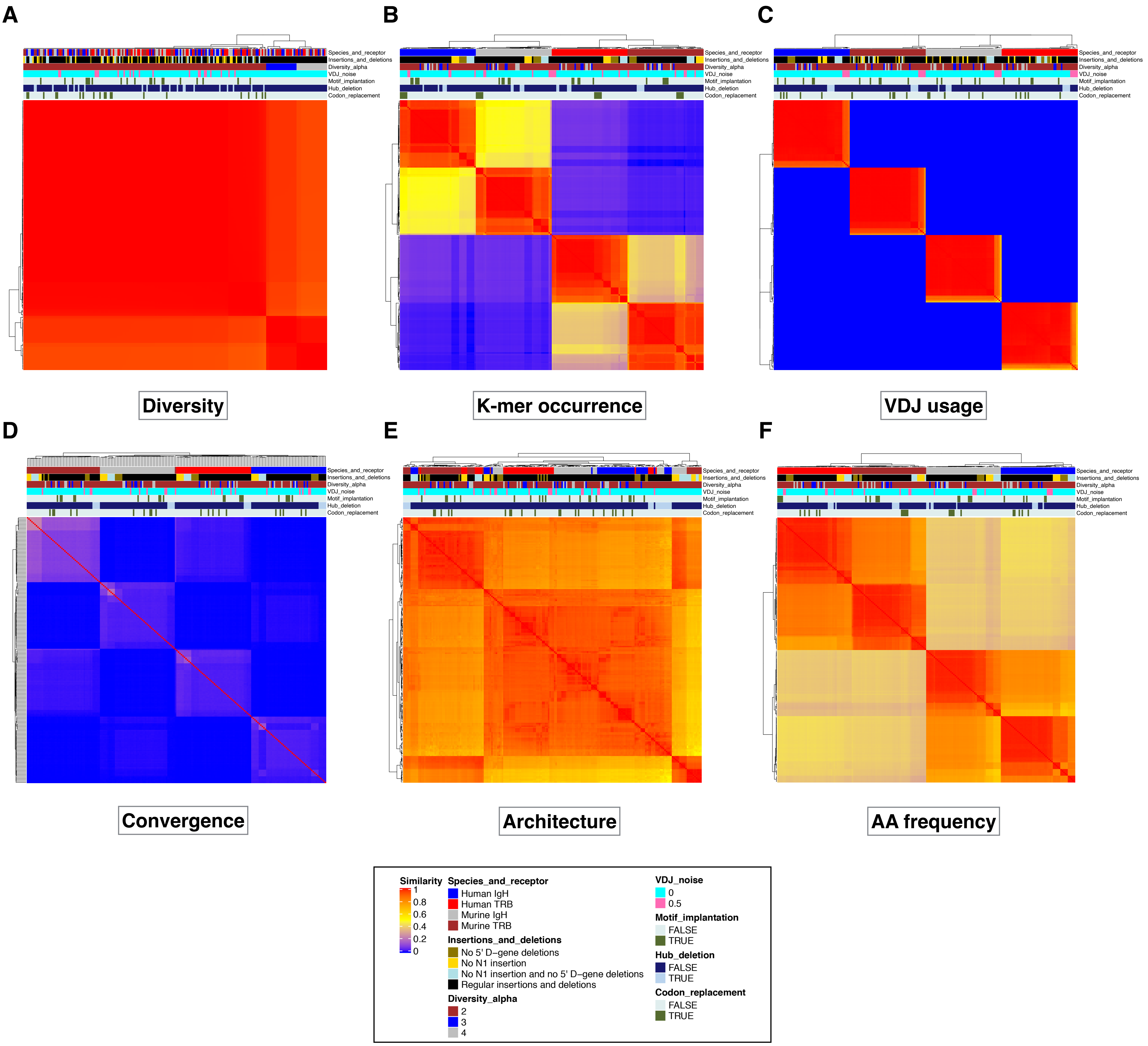

Supplementary Figure 1: Each single feature of the similarity network shows a unique topology, Related to Figure 2. (A–F) Heatmaps represent the similarity score landscape for each of the six features.

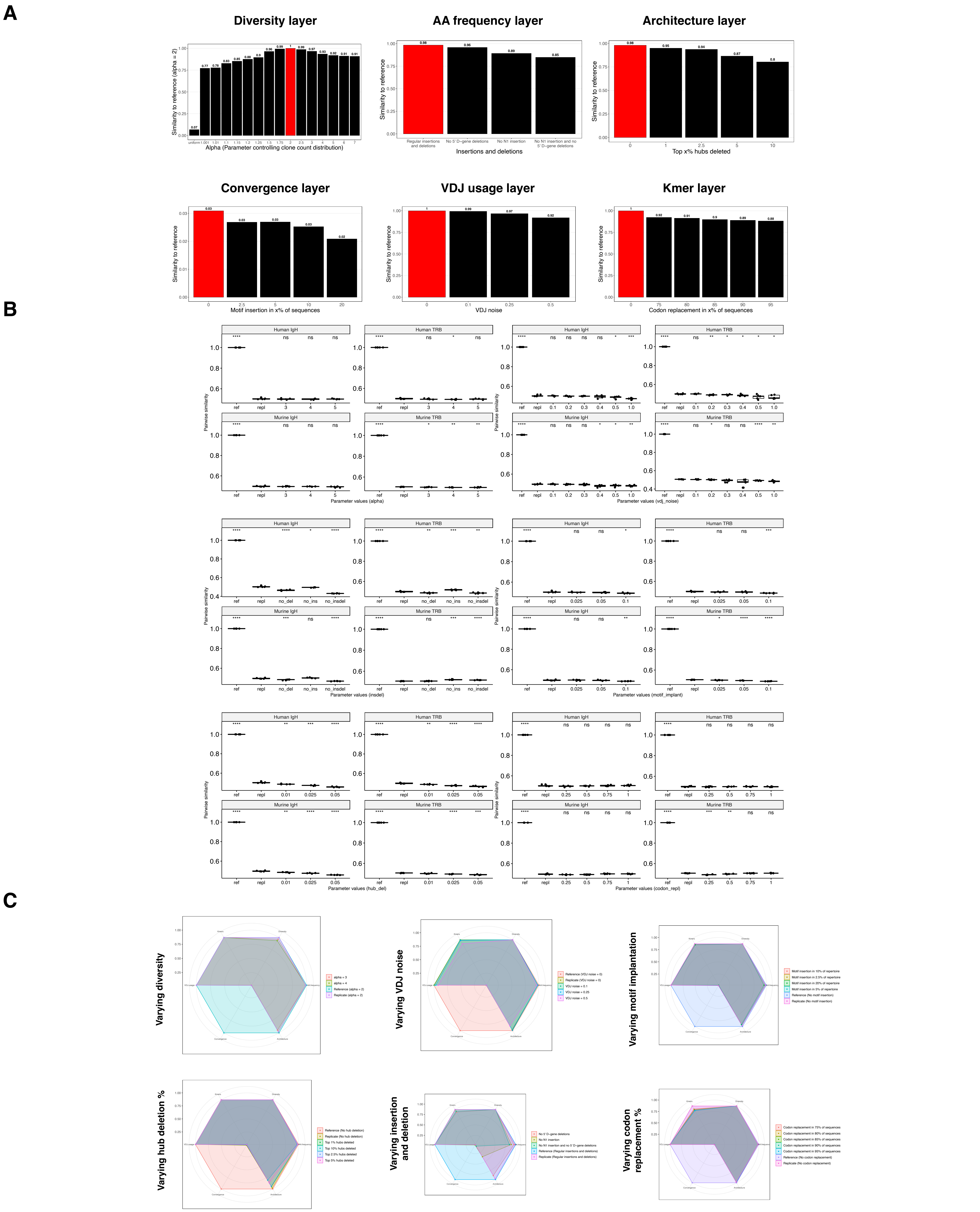

Supplementary Figure 2 I The similarity landscape of simulated repertoires defines a reference map for immune repertoire similarity. Related to Figure 2. (A) Per feature differences for a range of immuneSIM parameters (red bar indicates default parameters) (ns: $p > 0.05$, *: $p <= 0.05$, **: $p <= 0.01$, ***: $p <= 0.001$, ****: $p <= 0.0001$) **(B)** Similarities in each feature across different parameters (see also Fig 3A) (**(C)** Sensitivity of condensed similarity scores (y-axis) in response to different parameters (x-axis) faceted by species/receptor combination.

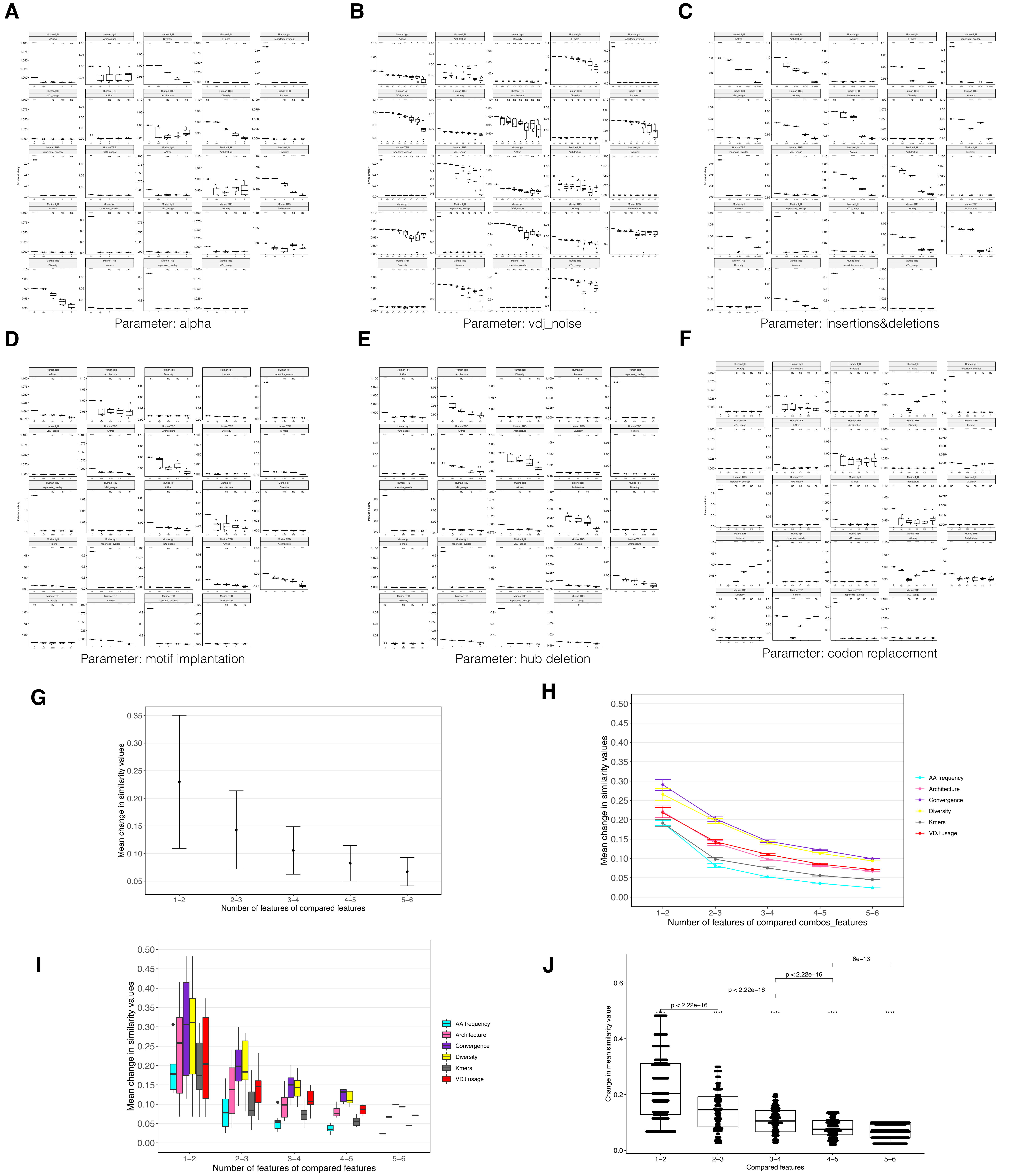

Supplementary Figure 3 I Sensitivity analysis per feature demonstrates the impact of each parameter change is feature specific and diminishing marginal utility of additional features Related to Figure 2. (A–F) The impact on pairwise similarity scores (y-axis) of parameter changes (x-axis) is shown for each parameter. Faceted by species/ receptor combination and feature. **(G)** The mean change in similarity values after the addition of the next similarity feature (feature to be added is chosen randomly, 500 iterations) **(H)** The mean change in similarity values for the addition of each feature during various stages of the construction of the multi-feature network. **(I)** Boxplots of mean change in similarity values for the addition of each feature during various stages of constructing a multi-feature network. **(J)** Beeswarm representation of Fig. 2D. Error bars show mean ± standard deviation across iterations. Significance values are defined as ns: $p > 0.05$, *: $p <= 0.05$, **: $p <= 0.01$, ***: $p <= 0.001$, ****: $p <= 0.0001$.

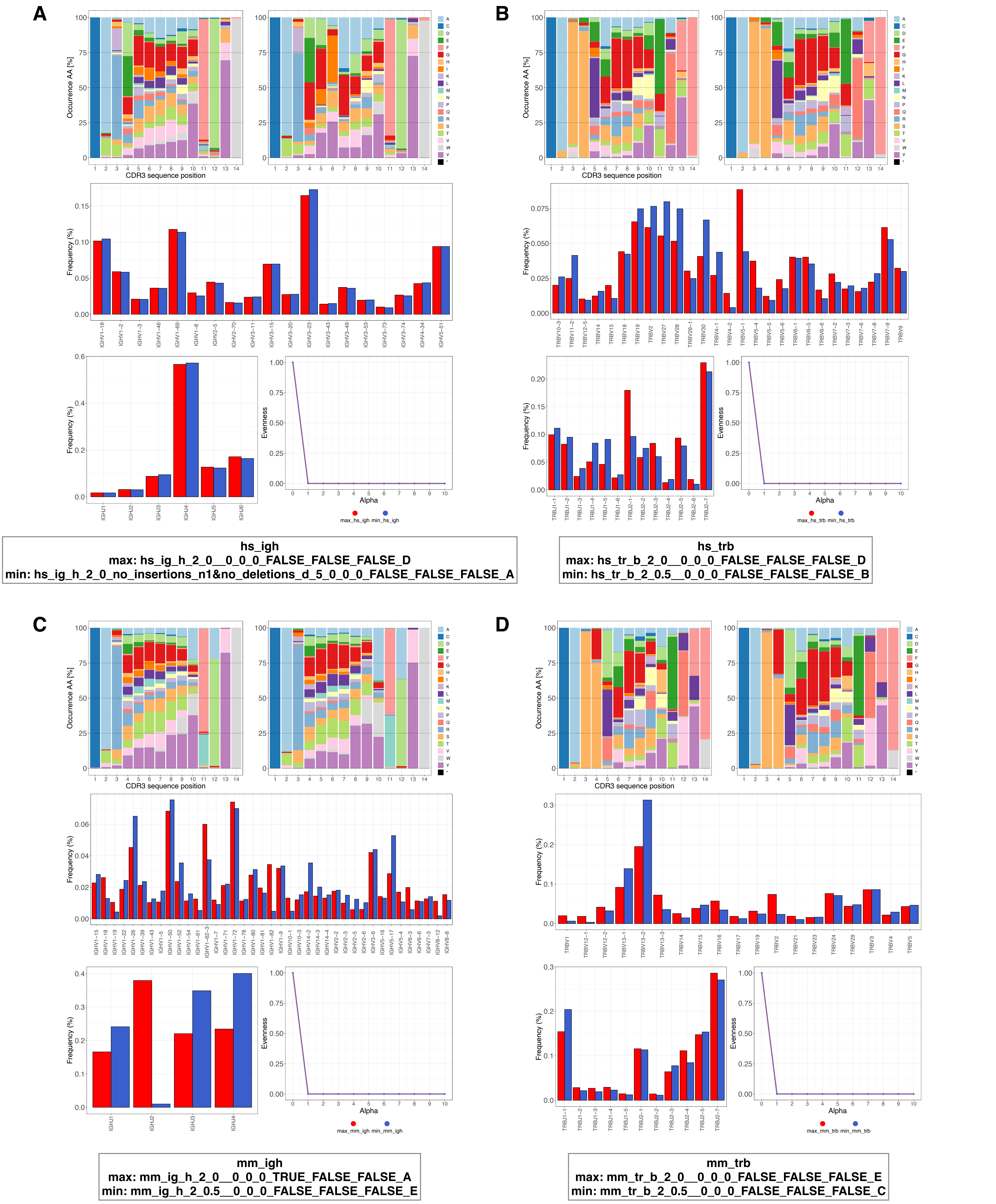

Supplementary Figure 4 | Within-cohort variability of simulated repertoires varies by receptor cohort (human and murine simulated repertoires). Related to Figure 3. The amino acid frequency (first row), VJ usage (middle row, bottom left), and evenness (bottom right) are compared between the most and least locally similar repertoires of human (hs) **(A)** IgH and **(B)** TRB simulated repertoires as well as mouse (mm) **(C)** IgH and **(D)** TRB simulated repertoires.

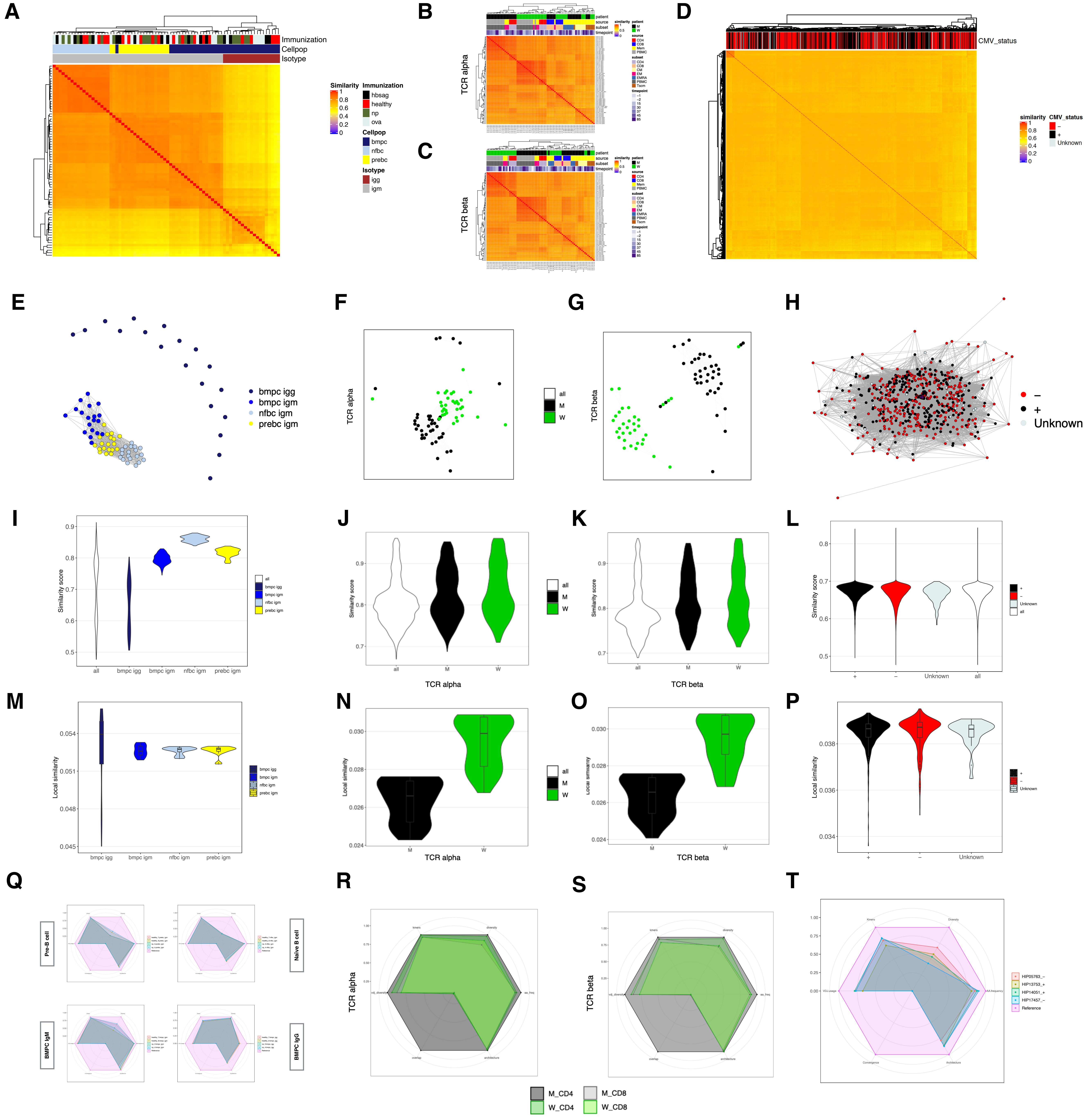

Supplementary Figure 5 I Application of immuneREF to 76 experimental repertoires from mouse immunization study (Greiff et al. 2017a), 72 T-cell repertoires from various cell populations of two Covid patients (longitudinal) and 666 experimental, CMV-serotyped repertoires (human, TCR, Emerson et al., 2017) finds even similarity distribution across repertoires of CMV+ and CMV- status. Related to Figure 4. (A) Similarity landscape of experimental (murine, BCR) repertoires from four immunization cohorts (Healthy, HBsAg, NP, OVA) and four cell populations (pre-B cell IgM, naïve B-cell IgM, bone marrow plasma cell IgM and IgG), **(B,C)** experimental (human, TCR, alpha and beta) repertoires for four cell populations **(D)** experimental (human, TCR) repertoires of CMV+ (289), CMV- (352) and unknown serotype (25) **(E–H)** Network visualizations of repertoires (nodes) and weighted edges between them representing similarity scores (top 25% edge by edge weights). **(I–L)** Distribution of similarity scores across the entire network and per cell population/condition shows variation within and across cohort. **(M–P)** Distribution of local similarity values per repertoire for each cell population/condition. **(Q–T)** Comparison of most locally similar repertoires of the conditions per cell population and immuneSIM reference repertoires (Reference, standard immuneSIM parameters).

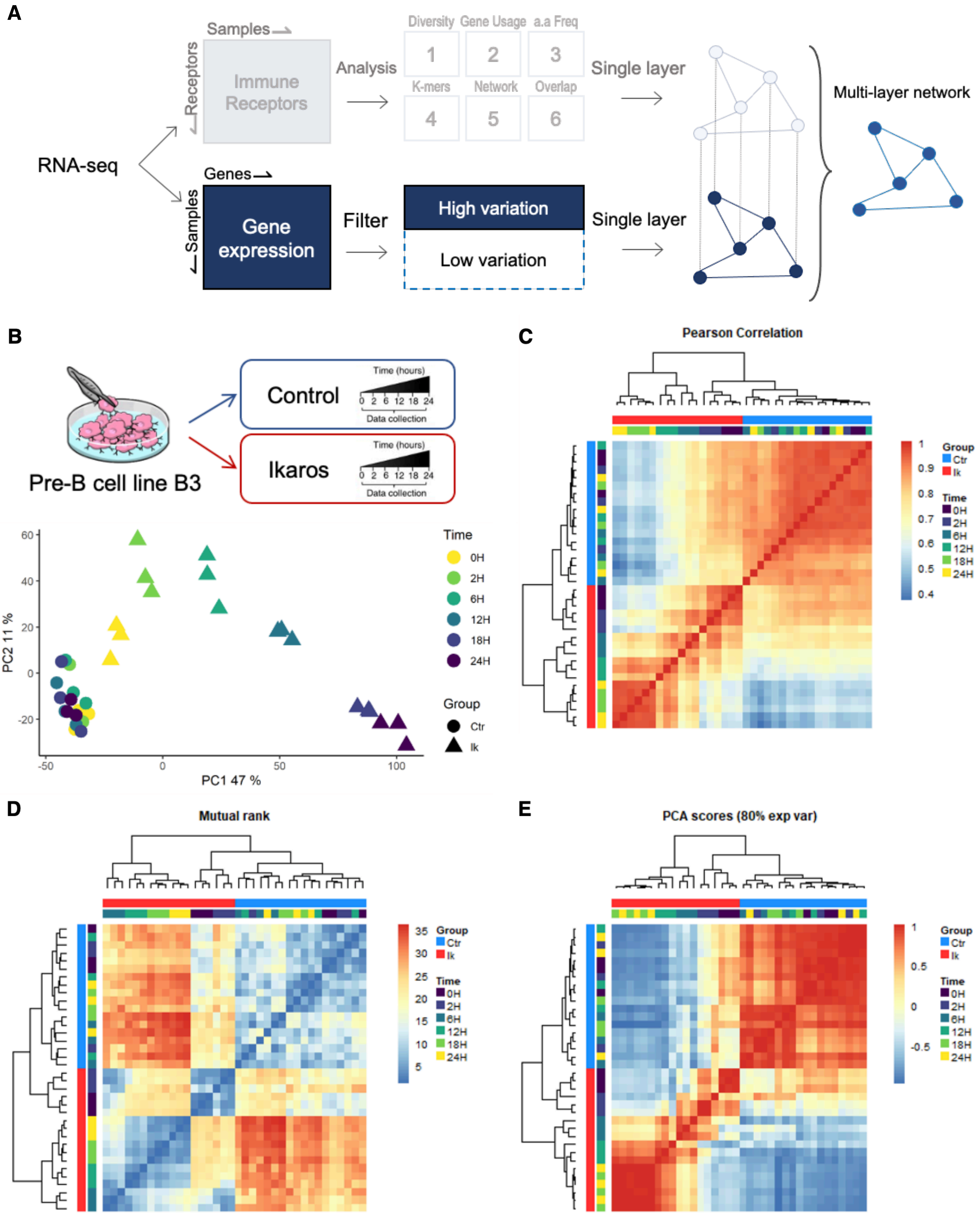

Supplementary Figure 6 I Gene expression and immune repertoire integration. Related to Figure 4. Gene expression can be added as an additional single feature to obtain the multi-feature network. **(A)** Pipeline overview. **(B)** Example of analysis using STATegra dataset (mouse pre-B cell line B3, RNA-seq, Gomez-Cabrero et al. 2019). **(C–E)** Heatmap representing co-expression patterns between samples using three methods available in the immuneREF package (Pearson Correlation, Mutual rank and PCA score).

Supplementary Table 1. Parameters for simulated datasets & hypothesized impact

**Parameter**	**Values/range**	**Hypothesized Impact (Feature)**
Alpha (clone count distribution)	**2**, 3, 4	Diversity feature only
Species	Human (hs), Mouse (mm)	All except diversity
Receptor chain	IgH, TRB	All except diversity
VDJ noise<br>(introduces noise into germline gene frequencies)	**0**, 0.5	Germline gene usage, kmer, A.A. frequency, Architecture
Insertion Deletion Dropout<br>(modifies inclusion of insertion and deletions)	**ins_and_del**, no_ins_n1, no_del_d_5, no_insertions_n1&no_deletions_d_5	Germline gene usage, kmer, A.A. frequency, Architecture
Motif implantation	True (implant "YAY" and "YVY" at a random position in 2.5% of sequences each), **False**	Convergence, (minor: architecture)
Hub deletion	True (Top 0.5 %), **False**	Architecture, Convergence
Synonym codon introduction	True ("tac" for "tat", "ggg" for "ggt" and "agc" for "agt", in 50% of sequences), **False**	K-mer, (minor: Convergence)

Supplementary Table 1 I ImmuneSIM parameters for simulated datasets & hypothesized impact. Related to Figure 2. The repertoires were simulated to differ across eight parameters. (In bold: Default parameters.) Please refer to immuneSIM documentation (https://immunesim.readthedocs.io/en/latest/parameters.html) for parameter definitions.

**Supplementary Table 2. Quantitative description of high-throughput sequencing datasets.**		
**Data Origin**	**Cell Type**	**No. of unique CDR3 sequences (mean±std.error)**
Mouse (C57BL/6J), B cell (Greiff et al., 2017a)	preBC (IGM) (19)<br>nBC (IGM) (19)<br>PC (IGM) (19)<br>PC (IGG) (19)	172’262 ± 9’394<br>395’558 ± 18’341<br>28’762 ±11’443<br>147 ± 18
Human (CMV-dataset), T cell (Emerson et al., 2017)	PBMC (CMV positive) (289)<br>PBMC (CMV negative) (351)<br>PBMC (CMV unknown) (26)	177’095 ± 4’235<br>186’951 ± 4’029<br>154’478 ± 14386
Human (Covid-dataset), T cell (TCRbeta) (Minervina et al., 2021)	CD4 (mild Covid) (10)<br>CD8 (mild Covid) (10)<br>Mem (mild Covid) (28)<br>PBMC (mild Covid) (24)	321’827 ± 34’100<br>189’289 ± 29’240<br>36’233 ± 6’232<br>623’003 ± 46’519
Human (Covid-dataset), T cell (TCRalpha) (Minervina et al., 2021)	CD4 (mild Covid) (10)<br>CD8 (mild Covid) (10)<br>Mem (mild Covid) (28)<br>PBMC (mild Covid) (24)	233’948 ± 32’085<br>141’119 ± 20’064<br>29’073 ± 5’253<br>425’560 ± 28’554
Human (BGI), T cell (Zhang et al., 2019)	PBMC (Healthy) (439))<br>PBMC (RA) (206)<br>PBMC (SLE) (877)	74’195 ± 25’364<br>123’292 ± 68’630<br>84’582 ± 52’944

Supplementary Table 2 I Quantitative description of high-throughput sequencing datasets. Related to Figure 4. Overview of the number of unique CDR3 sequences of the experimental datasets analyzed using immuneREF. Where the number of clonal sequences exceeded 10’000, the immune repertoire was subsampled by extracting the 10’000 clonal sequences with the highest clone count.
